# Flexible Bayesian estimation of incubation times

**DOI:** 10.1093/aje/kwae192

**Published:** 2024-07-10

**Authors:** Oswaldo Gressani, Andrea Torneri, Niel Hens, Christel Faes

**Affiliations:** Interuniversity Institute for Biostatistics and Statistical Bioinformatics (I-BioStat), Data Science Institute Hasselt University, Hasselt BE-3500, Belgium; Interuniversity Institute for Biostatistics and Statistical Bioinformatics (I-BioStat), Data Science Institute Hasselt University, Hasselt BE-3500, Belgium; Interuniversity Institute for Biostatistics and Statistical Bioinformatics (I-BioStat), Data Science Institute Hasselt University, Hasselt BE-3500, Belgium; Centre for Health Economics Research and Modelling Infectious Diseases, Vaxinfectio, University of Antwerp, Antwerp BE-2000, Belgium; Interuniversity Institute for Biostatistics and Statistical Bioinformatics (I-BioStat), Data Science Institute Hasselt University, Hasselt BE-3500, Belgium

**Keywords:** incubation period, Laplace approximation, Bayesian P-splines, MCMC

## Abstract

The incubation period is of paramount importance in infectious disease epidemiology as it informs about the transmission potential of a pathogenic organism and helps the planning of public health strategies to keep an epidemic outbreak under control. Estimation of the incubation period distribution from reported exposure times and symptom onset times is challenging as the underlying data is coarse. We developed a new Bayesian methodology using Laplacian-P-splines that provides a semiparametric estimation of the incubation density based on a Langevinized Gibbs sampler. A finite mixture density smoother informs a set of parametric distributions via moment matching and an information criterion arbitrates between competing candidates. Algorithms underlying our method find a natural nest within the EpiLPS package, which has been extended to cover estimation of incubation times. Various simulation scenarios accounting for different levels of data coarseness are considered with encouraging results. Applications to real data on coronavirus disease 2019, Middle East respiratory syndrome, and Mpox reveal results that are in alignment with what has been obtained in recent studies. The proposed flexible approach is an interesting alternative to classic Bayesian parametric methods for estimation of the incubation distribution.

## Introduction

Statistical methods and their underlying algorithmic implementation play an essential role in infectious disease modeling as they permit investigators to bridge the gap between observed data and estimates of key epidemiologic quantities. The incubation period, defined as the time between infection and symptom onset,^1^ is pivotal in gauging the epidemic potential of an infectious disease. Having information about the incubation period distribution is helpful for planning optimal quarantine periods to taper off the spread of a contagious disease.^2^ Moreover, incubation times help in assessing the transmission potential of an infectious disease as they are key components in estimating the distribution of generation time, which in turn can be used to estimate the reproduction number.^3^^,^^4^ The incubation period is also of direct interest for case definition^5^ and to measure the effectiveness of contact tracing.

From a statistical point of view, the main obstacle for inferring the distribution of the incubation period lies in the fact that infection times are almost never exactly observed,^6^ while symptom onset times are more easily observed and reported. This incomplete information setup pushes towards a more challenging inference approach based on coarse data,^7^ where infection times are only known to lie within a finite time interval. The work of Reich et al^7^ proposes frequentist parametric approaches to estimate the incubation period distribution using the accelerated failure time model with applications to influenza A and RSV. Backer et al^8^ and Miura et al^9^ use a Bayesian parametric approach to estimate the incubation period of COVID-19 and of Mpox, respectively. Groeneboom^10^ derives a smooth nonparametric estimator of the incubation time distribution by adding a bandwidth parameter that controls the trade-off between noise and bias, Kreiss and Van Keilegom^11^ propose a semiparametric method to estimate the incubation period based on Laguerre polynomials.

The current trend in applied papers aiming at estimating the incubation period of an infectious disease is to rely on parametric models. Although mathematically appealing, the main shortcoming of working with standard parametric families is the risk of missing important features in epidemic data.^10^ The central importance of the incubation period in epidemic analyses has motivated our aim to develop a flexible methodology that is not limited by the boundaries imposed by parametric assumptions. We thus propose a new semiparametric Bayesian approach to estimate the incubation period distribution articulated around Laplacian-P-splines (LPS).^12^^,^^13^

Our methodology is an interesting alternative to fully parametric schemes in the sense that the best fitting incubation distribution is selected in a data-driven way by automatically choosing between a semiparametric fit and a candidate coming from popular parametric families. As such, the proposed tool may be useful for researchers or public health officers aiming to obtain flexible estimates of the incubation period distribution based on exposure information and symptom onset data.

## Methods

### Coarsely observed data

**Figure 1:**
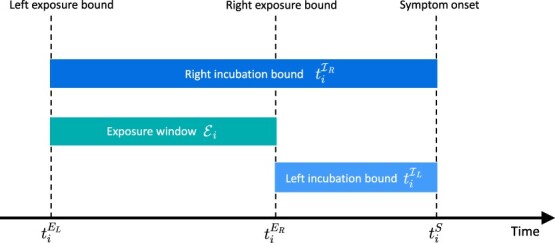
Relationship between exposure times, incubation bounds, and the symptom onset time for an infectious disease.

The observed symptom onset time for individual $i$ is denoted by ${t}_i^S$ and the (unobserved) infection time is only known to lie within the closed exposure interval ${\mathcal{E}}_i=\left[{t}_i^{E_L},{t}_i^{E_R}\right]$, where ${t}_i^{E_L}$ and ${t}_i^{E_R}$ denote the left and right bound, respectively, of the infecting exposure time. Without loss of generality, we work from a continuous time perspective and assume that $0\le{t}_i^{E_L}<{t}_i^{E_R}<{t}_i^S$ and that symptom onset times are finite. The incubation time is thus at least ${t}_i^{{\mathcal{I}}_L}={t}_i^S-{t}_i^{E_R}$ and at most ${t}_i^{{\mathcal{I}}_R}={t}_i^S-{t}_i^{E_L}$, so that the observed data at the resolution of individual $i$ is given by the bounds of the incubation period ${\mathcal{D}}_i=\left\{{t}_i^{{\mathcal{I}}_L},{t}_i^{{\mathcal{I}}_R}\right\}$ and the information of an observable set of size $n$ is thus $\mathcal{D}={\cup}_{i=1}^n{\mathcal{D}}_i$. Figure 1 gives a graphical illustration of the relationship between exposure times, incubation bounds, and the symptom onset time for individual $i$.

### Semiparametric model with Bayesian P-splines

Let the incubation time $\mathcal{I}$ be a nonnegative continuous random variable with probability density function $\varphi \left(\cdotp \right)$, hazard function $h\left(\cdotp \right)$ and survival function $S\left(\cdotp \right)$. Based on a dataset $\mathcal{D}$, we propose to estimate $\varphi \left(\cdotp \right)$ by a two-component mixture density using a semiparametric (SP) approach based on P-splines.^14^ The candidate density estimator at a given time point $t\ge 0$ is denoted by ${\hat{\varphi}}_{SP}(t)=\omega{\hat{\varphi}}_{IC}(t)+\left(1-\omega \right){\hat{\varphi}}_{HS}(t)$, with $0\le \omega \le 1$. The density estimator ${\hat{\varphi}}_{IC}\left(\cdotp \right)$ is based on single interval-censored IC data as shown in Figure 1, while ${\hat{\varphi}}_{HS}\left(\cdotp \right)$ is a density estimator resulting from a histogram smoother (HS) assuming a midpoint imputation rule for the infection time in the exposure window $\mathcal{E}$.

### Flexible density estimation for single interval-censored data

Following Rosenberg,^15^ the (log-)hazard of the incubation period is approximated by a linear combination of (cubic) B-spline basis functions:


(1) \begin{equation*} \log h(t)=\sum_{k=1}^K{\theta}_k{b}_k(t), \end{equation*}

where $b\left(\cdotp \right)={\left({b}_1\left(\cdotp \right),\dots, {b}_K\left(\cdotp \right)\right)}^{\top }$ is a B-spline basis having equidistant knots on the compact time interval $\mathcal{T}=\left[0,{t}_u\right]$ with upper bound ${t}_u$, and $\boldsymbol{\theta} ={\left({\theta}_1,\dots, {\theta}_K\right)}^{\top }$ is the $K$-dimensional latent vector of B-spline amplitudes. While zero is a natural lower bound for the incubation period, there is no natural choice for the upper bound ${t}_u$. An intuitive candidate would be to fix it at the largest observed right bound of the incubation time, ie, ${t}_u=\max \left\{{t}_1^{{\mathcal{I}}_R},\dots, {t}_n^{{\mathcal{I}}_R}\right\}$; however, the latter choice may restrict the B-spline basis to a domain that covers only a small part of the domain of the true underlying incubation density $\varphi \left(\cdotp \right)$. As such, we follow Eilers and Marx^16^ and advise padding ${t}_u$ to a value that is strictly larger than the largest observed incubation bound. We defer the discussion on the guidelines for a smart padding choice to the real data applications section. Regarding the number $K$ of B-spline basis functions, a default choice in the present setting is $K=10$, although larger numbers may be necessary to capture more flexible patterns. As noted by Eilers and Marx,^16^ there is no fear to choose “too large” a number $K$, as the penalty will act as a counterforce to the induced flexibility. Using the relation between the survival and hazard functions, we recover:


$$ S(t)=\exp \left(-{\int}_0^th(s) ds\right)\kern0.5em \mathrm{and} $$

(2) \begin{equation*} \overset{\sim }{S}(t)\approx \exp \left(-\sum_{j=1}^{j(t)}\exp \left({\boldsymbol{\theta}}^{\top }b\left({s}_j\right)\right)\Delta \right). \end{equation*}

The approximation in equation 2 is necessary as the integral has no analytical solution. As such, $\mathcal{T}$ is partitioned into a large number of bins $J$ (eg, $J=300$) having equal width $\Delta$, where ${s}_j$ denotes the center of the $j$th bin and $j(t)\in \left\{1,\dots, J\right\}$ is an index function returning the bin number containing $t$. Following Lang and Brezger,^17^ a zero-mean Gaussian prior is imposed on the vector of B-spline amplitudes $\boldsymbol{\theta} \mid \lambda \sim{\mathcal{N}}_{\dim \left(\boldsymbol{\theta} \right)}\left(0,{\left(\lambda P\right)}^{-1}\right)$, where $\lambda >0$ is the penalty parameter related to the spline model, and $P={D}_r^{\top }{D}_r+\varepsilon{I}_{\dim \left(\boldsymbol{\theta} \right)}$ is a square penalty matrix obtained from $r$th order difference matrices ${D}_r$ of dimension $\left(\dim \left(\boldsymbol{\theta} \right)-r\right)\times \dim \left(\boldsymbol{\theta} \right)$, perturbed by an $\varepsilon$-multiple (here $\varepsilon =1{0}^{-6}$) to ensure $P$ is full rank. The Bayesian model is closed by assuming a noninformative Gamma prior on the penalty parameter $\lambda \sim \mathcal{G}\left({a}_{\lambda },{b}_{\lambda}\right)$ with shape ${a}_{\lambda }=1{0}^{-4}$ and rate ${b}_{\lambda }=1{0}^{-4}.$^18^^,^^19^ The likelihood of incubation times under single interval-censored data is^7^:


$$ \mathcal{L}\left(\boldsymbol{\theta}; \mathcal{D}\right)=\prod_{i=1}^n\left({\int}_{t_i^{{\mathcal{I}}_L}}^{t_i^{{\mathcal{I}}_R}}\varphi (t) dt\right)=\prod_{i=1}^n\left(S\left({t}_i^{{\mathcal{I}}_L}\right)-S\left({t}_i^{{\mathcal{I}}_R}\right)\right). $$

Replacing $S\left(\cdotp \right)$ with ${\widetilde S}\left(\cdotp \right)$ yields:


\begin{align*} \mathcal{L}\left(\boldsymbol{\theta}; \mathcal{D}\right)&=\prod_{i=1}^n\left(\exp \left(-\sum_{j=1}^{j\left({t}_i^{{\mathcal{I}}_L}\right)}\exp \left({\boldsymbol{\theta}}^{\top }b\left({s}_j\right)\right)\Delta \right)\right.\nonumber\\&\left.\quad-\exp \left(-\sum_{j=1}^{j\left({t}_i^{{\mathcal{I}}_R}\right)}\exp \left({\boldsymbol{\theta}}^{\top }b\left({s}_j\right)\right)\Delta \right)\right), \end{align*}

where the equality sign is kept for notational convenience and $\mathcal{L}\left(\boldsymbol{\theta}; \mathcal{D}\right)$ is contemplated from here on as an approximate version of the likelihood function. The (approximate) log-likelihood is:


$$ \ell \left(\boldsymbol{\theta}; \mathcal{D}\right):= \log \mathcal{L}\left(\boldsymbol{\theta}; \mathcal{D}\right) $$

(3) \begin{align*} &=\sum_{i=1}^n\log \left(\exp \left(-\sum_{j=1}^{j\left({t}_i^{{\mathcal{I}}_L}\right)}\exp \left({\boldsymbol{\theta}}^{\top }b\left({s}_j\right)\right)\Delta \right)\right.\notag\\ &\left.\quad-\exp \left(-\sum_{j=1}^{j\left({t}_i^{{\mathcal{I}}_R}\right)}\exp \left({\boldsymbol{\theta}}^{\top }b\left({s}_j\right)\right)\Delta \right)\right). \end{align*}

From Bayes' theorem, one obtains the (log-)conditional posterior density:


$$ p\left(\boldsymbol{\theta} |\lambda, \mathcal{D}\right)\propto \exp \left(\ell \left(\boldsymbol{\theta}; \mathcal{D}\right)\right)p\left(\boldsymbol{\theta} |\lambda \right) $$

$$ \propto \exp \left(\ell \left(\boldsymbol{\theta}; \mathcal{D}\right)-\frac{\lambda }{2}{\boldsymbol{\theta}}^{\top }P\boldsymbol{\theta} \right) $$

(4) \begin{equation*} \log p\left(\boldsymbol{\theta} |\lambda, \mathcal{D}\right)\dot{=}\ell \left(\boldsymbol{\theta}; \mathcal{D}\right)-\frac{\lambda }{2}{\boldsymbol{\theta}}^{\top }P\boldsymbol{\theta}, \end{equation*}

where $\propto$ and $\dot{=}$ are symbols used to denote equality up to a multiplicative and additive constant, respectively. The Laplace approximation to the conditional posterior of the B-spline amplitudes is obtained by fitting a (multivariate) Gaussian density around the mode of $p\left(\boldsymbol{\theta} |\lambda, \mathcal{D}\right)$. This permits the analyst to recover the Laplace approximation ${\tilde{p}}_G\left(\boldsymbol{\theta} |{\lambda}^{\ast },\mathcal{D}\right)={\mathcal{N}}_{\dim \left(\boldsymbol{\theta} \right)}\left({\boldsymbol{\theta}}^{\ast}\left({\lambda}^{\ast}\right),{\Sigma}^{\ast}\left({\lambda}^{\ast}\right)\right)$, where ${\lambda}^{\ast }$ is a maximum a posteriori estimate of the penalty parameter (see Appendix S1 for details). The Laplace approximation and gradient of the log-likelihood are used in the Langevinized Gibbs sampler (LGS) developed in Gressani et al^20^ to sample from the joint posterior of the model parameters $p\left(\boldsymbol{\theta}, \lambda |\mathcal{D}\right)$, and the point estimate (posterior median) of $\boldsymbol{\theta}$ is denoted by $\hat{\boldsymbol{\theta}}$. Plugging the latter into the formulas of the hazard in equation 1 and the survival in equation 2, we obtain the point estimates $\hat{h}(t)$ and $\hat{\overset{\sim }{S}}(t)$ at a given time point $t$. Finally, exploiting the relation between the density, the hazard, and the survival functions, our semiparametric estimate of the incubation density based on interval-censored data is ${\hat{\varphi}}_{IC}(t)=\hat{h}(t)\hat{\overset{\sim }{S}}(t)\kern0.5em \forall t\ge 0$.

### Flexible density estimation for midpoint imputation

The second component of the mixture density estimator ${\hat{\varphi}}_{HS}\left(\cdotp \right)$ under the semiparametric approach is obtained through a midpoint imputation technique. Starting from the incubation bounds in $\mathcal{D}$, we construct an artificial dataset $\overset{\sim }{\mathcal{D}}=\left\{\overset{\sim }{t_i}:i=1,\dots, n\right\}$, where the infection time of individual $i$ is assumed to be located in the middle of the incubation interval, so that the imputed incubation time is:


$$ \overset{\sim }{t_i}=0.5\left({t}_i^{{\mathcal{I}}_L}+{t}_i^{{\mathcal{I}}_R}\right) $$

$$ =0.5\left({t}_i^S-{t}_i^{E_R}+{t}_i^S-{t}_i^{E_L}\right) $$

$$ ={t}_i^S-0.5\left({t}_i^{E_L}+{t}_i^{E_R}\right). $$

Note that $\overset{\sim }{\mathcal{D}}$ is seen as a random sample from the incubation density $\varphi \left(\cdotp \right)$. From ideas in Eilers and Marx,^21^ we construct a histogram on the time domain $\overset{\sim }{\mathcal{T}}=\left(0,{\tilde{t}}_u\right]$ and recommend using an upper bound that is at least equal to ${t}_u$, ie,. ${\tilde{t}}_u\ge{t}_u$. $\overset{\sim }{\mathcal{T}}$ is partitioned in $L$ bins with midpoint ${x}_l$ and width $h$ so that the $l$th bin is the half-open interval ${\mathcal{B}}_l=\left({x}_l-h/2,{x}_l+h/2\right]$. Typically, the histogram smoother is not very sensitive to the choice of the binwidth, provided narrow bins (eg, $h=0.05$) are used.^22^ Another possibility is to use a binwidth $h$ determined by a preliminary kernel smoother. The number of imputed incubation periods falling in bin $l$ is ${y}_l={\sum}_{i=1}^n\mathbb{I}\left({\tilde{t}}_i\in{\mathcal{B}}_l\right)$, where $\mathbb{I}\left(\cdotp \right)$ is the indicator function. The count variable ${y}_l$ is assumed to follow a negative binomial distribution ${y}_l\sim \mathrm{NegBin}\left({\mu}_l,\rho \right)$ with mean ${\mu}_l>0$ and overdispersion parameter $\rho >0$. We impose a cubic B-spline basis on $\overset{\sim }{\mathcal{T}}$ and model the log of the mean counts as $\log \left({\mu}_l\right)={\sum}_{k=1}^K{\theta}_k{b}_k\left({x}_l\right)$. The beauty behind such a formulation is that it allows us to recover exactly the same model as in EpiLPS^20^ to smooth case counts. We thus refer the reader to the latter reference to consult all the equations related to the Laplacian-P-splines approach leading to an estimate of the vector of B-spline coefficients $\hat{\boldsymbol{\theta}}$. The density estimate resulting from histogram smoothing is then given by: ${\hat{\varphi}}_{HS}(t)={(nh)}^{-1}\exp \left({\sum}_{k=1}^K{\hat{\theta}}_k{b}_k(t)\right)\kern0.5em \forall t\ge 0$ and assuming equal weights $\omega =0.5$, our semiparametric mixture density estimator for the incubation density $\varphi (t)$ at a given time point $t\ge 0$ is ${\hat{\varphi}}_{SP}(t)=0.5\left({\hat{\varphi}}_{IC}(t)+{\hat{\varphi}}_{HS}(t)\right)$.

### Parametric fits using moment matching

In some situations it may be advantageous to fit the data by using well-known parametric distributions. Our methodology leaves a door open for this possibility by informing three classic parametric distributions that are usually considered in the estimation of the incubation period, namely the two-parameter lognormal, Gamma, and Weibull families. The moment matching approach to fit the latter distributions is given in Appendix S2.

### Simulation settings

To assess the performance of our methodology, we designed various simulation scenarios with different target incubation densities (Table 1), data coarseness, and sample size. We assumed two levels of data coarseness, with average exposure window $\mathcal{E}$ equal to 1 or 2 days and exposure windows with maximum width of 7 days, reflecting a range that is often observed in practice.^23^ For the sample size, we fix $n=40$ and $n=100$, to see how our method performs under small and medium sample size. In Appendix S3, additional results are provided for $n=200$ (Tables S1-S5). The features on which we assess the performance are the mean and standard deviation of the incubation period and additional percentiles that are typically of particular interest (eg, the 5th, 50th, and 95th percentiles). It turns out that for most infectious diseases, incubation times have a tendency to be well approximated by a lognormal distribution.^1^^,^^24^ This motivates our choice to include the latter incubation distribution as a target in the data-generating mechanism as well as the Weibull and Gamma, which are common choices^25^ as they can provide similar shapes to a lognormal density. To highlight the flexibility of our method, we also construct a flexible bimodal incubation density based on a mixture of two Weibull distributions that translates the presence of a cluster with longer incubation periods.^10^^,^^26^ Such bimodal patterns may arise when jointly analyzing epidemic data from different strains of a virus^27^ or when infectors and infectees do not share the same incubation period distribution.^6^ From the combination of all these settings, we obtain a total of $4\times 2\times 2=16$ scenarios. We also make a graphical evaluation of the fits by overlaying the density estimates with the target incubation density. Moreover, we are also interested in the performance of the selection process of our methodology, ie, how many times our approach selects the correct parametric family that corresponds to the incubation distribution used in the data-generating mechanism. For each scenario, we fix the number of replicated datasets following a common choice in the literature, namely $S=1000.$^28^^,^^29^

**Table 1:** Incubation distributions used in the data-generating mechanism of the simulation study.

**Reference**	**Distribution**	**Mean (days)**	**Standard deviation (days)**
Ferretti et al^30^	Lognormal	5.5	2.1
Backer et al^8^	Weibull	6.4	2.3
This study	Flexible bimodal	7.5	4.6
Donnelly et al^31^	Gamma	3.8	2.9

Such a number is large enough to assess how close (and at most how distant) our fitted incubation densities are to the target and at the same time allows the simulations to be replicated in a reasonable time limit on a standard computer. We use $K=10$ B-spline basis functions for all scenarios, except for the bimodal scenario, where $K=20$ to capture the more flexible density pattern. The number of Markov chain Monte Carlo iterations for the LGS sampler is fixed at $M=1000$, and the acceptance rate varied closely in the neighborhood of the optimal acceptance rate ($57\%$) in all scenarios.

## Results

**Figure 2:**
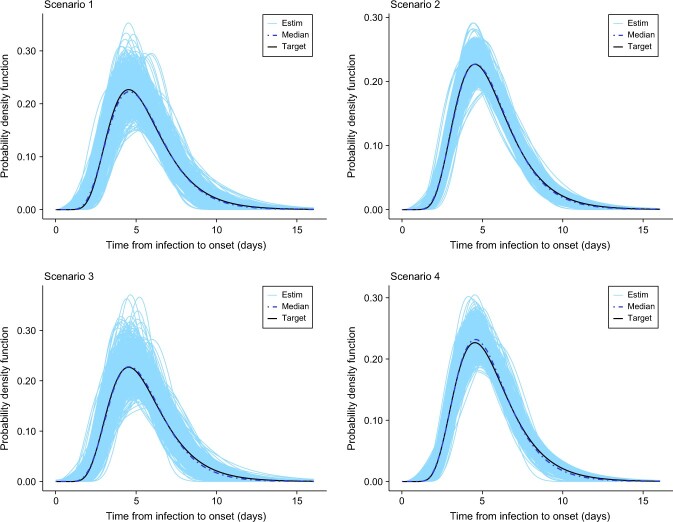
Estimated incubation densities for scenarios 1-4. The dash-dotted line is the pointwise median across the $S=1000$ simulations, and the solid black line is the lognormal incubation density from Ferretti et al.^30^

**Figure 3:**
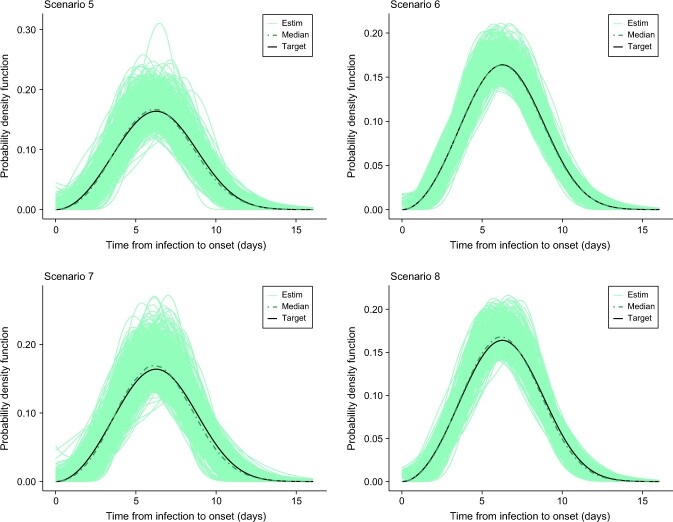
Estimated incubation densities for scenarios 5-8. The dash-dotted line is the pointwise median across the $S=1000$ simulations, and the solid black line is the Weibull incubation density from Backer et al.^8^

**Figure 4:**
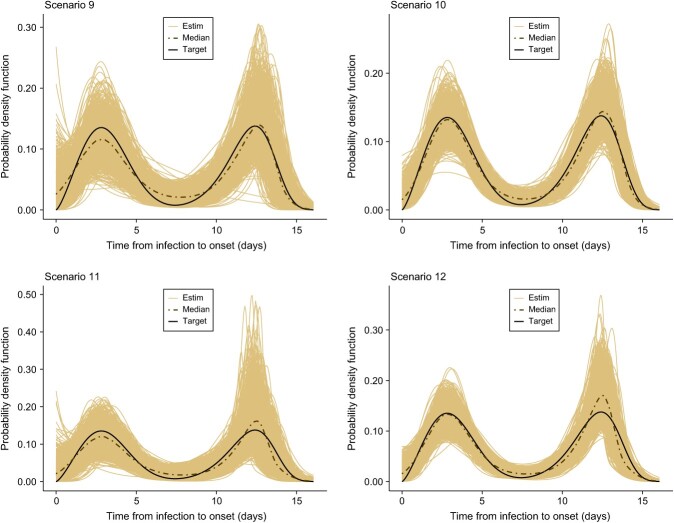
Estimated incubation densities for scenarios 9-12. The dash-dotted line is the pointwise median across the $S=1000$ simulations, and the solid black line is an artificial incubation density constructed as a mixture of two Weibull distributions.

**Figure 5:**
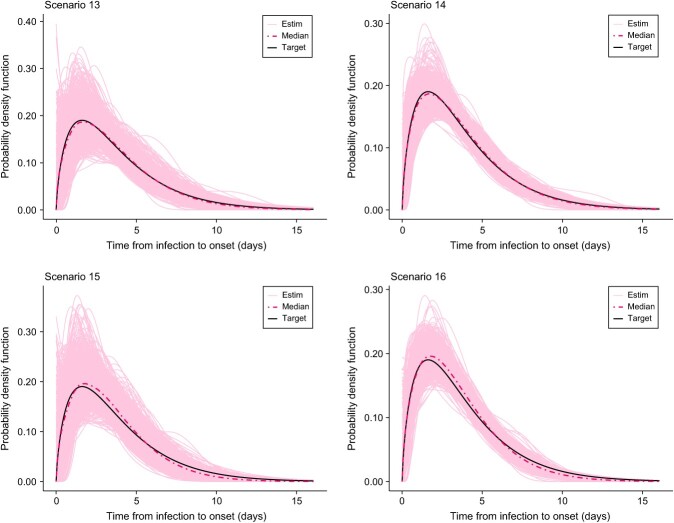
Estimated incubation densities for scenarios 13-16. The dash-dotted line is the pointwise median across the $S=1000$ simulations, and the solid black line is the Gamma incubation density from Donnelly et al.^31^

Tables 2-5 summarize the results for selected pointwise features of the incubation density for all scenarios (scenarios 1-16). In general, the bias is relatively small for all features but is more pronounced for the 95th percentile as less information is available in that region in the sense that fewer data points are collected in such a remote location of the domain of the incubation density. In addition, we observed that an increase in the sample size leads to a decrease in the root mean square error. From Figures 2-5, we see that, in general, the estimates provided by our method are able to nicely capture the target incubation densities. Thanks to the flexibility of our approach, even bimodal densities (Figure 4) are well reconstructed, which would not be feasible with parametric approaches relying on classic families. Moreover, the dash-dotted curves (representing the pointwise median of the estimates across the $S=1000$ simulated datasets) are in most cases not distinguishable from the target incubation density. Also, the fitted densities appear closer to the target with $n=100$ as compared with $n=40$ as more information is available. Finally, Table 6 shows that our method is quite efficient in detecting the true underlying distribution from which data is generated. For the lognormal incubation target, our LPS model selected the lognormal model in at least $73\%$ of cases with $n=100$ and at least $67\%$ of cases with $n=40$. A correct selection is even made in approximately $86\%$ of cases in the Weibull setting with $n=100$. Interestingly, our methodology almost never selects any parametric candidate when the underlying truth is a bimodal density. Although this may not be the case for lower sample sizes, it is still an encouraging sign. Finally, for the Gamma case, our model hesitates between a Gamma and a Weibull, but this is not really a problem as the main features of the true underlying Gamma density are still relatively well captured (see Table 5).

**Table 2:** Performance measures for selected features of the incubation density for two levels of data coarseness with $n=40$ and $n=100$.^a^

	**Average coarseness: 1 day**						
		$\boldsymbol{n}=\textbf{40}$ **(Scenario 1)**			$\boldsymbol{n}=\textbf{100}$ **(Scenario 2)**		
	**True**	**Average**	**Bias**	**RMSE**	**Average**	**Bias**	**RMSE**
Mean	5.528	5.471	−0.057	0.320	5.477	−0.052	0.208
SD	2.075	1.991	−0.084	0.300	1.993	−0.082	0.195
${q}_{0.05}$	2.849	2.800	−0.049	0.278	2.828	−0.021	0.174
${q}_{0.25}$	4.052	4.051	0.000	0.270	4.053	0.001	0.171
${q}_{0.50}$	5.176	5.177	0.002	0.312	5.169	−0.007	0.197
${q}_{0.75}$	6.612	6.564	−0.048	0.410	6.559	−0.053	0.262
${q}_{0.95}$	9.403	9.140	−0.264	0.831	9.170	−0.234	0.542
	**Average coarseness: 2 days**						
		$\boldsymbol{n}=\textbf{40}$ **(Scenario 3)**			$\boldsymbol{n}=\textbf{100}$ **(Scenario 4)**		
	**True**	**Average**	**Bias**	**RMSE**	**Average**	**Bias**	**RMSE**
Mean	5.528	5.439	−0.090	0.343	5.431	−0.097	0.225
SD	2.075	1.942	−0.133	0.315	1.942	−0.133	0.222
${q}_{0.05}$	2.849	2.818	−0.031	0.278	2.836	−0.013	0.179
${q}_{0.25}$	4.052	4.053	0.001	0.277	4.044	−0.008	0.173
${q}_{0.50}$	5.176	5.157	−0.018	0.328	5.138	−0.038	0.205
${q}_{0.75}$	6.612	6.511	−0.100	0.443	6.492	−0.120	0.287
${q}_{0.95}$	9.403	9.013	−0.391	0.888	9.022	−0.381	0.624

Abbreviations: RMSE, root mean square error; SD, standard deviation.

^a^ Results are for $S=1000$ simulated datasets and the lognormal incubation density from Ferretti et al.^30^

**Table 3:** Performance measures for selected features of the incubation density for two levels of data coarseness with $n=40$ and $n=100$.^a^

	**Average coarseness: 1 day**						
		$\boldsymbol{n}=\textbf{40}$ **(Scenario 5)**			$\boldsymbol{n}=\textbf{100}$ **(Scenario 6)**		
	**True**	**Average**	**Bias**	**RMSE**	**Average**	**Bias**	**RMSE**
Mean	6.403	6.369	−0.034	0.350	6.393	−0.010	0.229
SD	2.327	2.284	−0.043	0.246	2.325	−0.002	0.151
${q}_{0.05}$	2.665	2.767	0.102	0.504	2.690	0.026	0.312
${q}_{0.25}$	4.734	4.737	0.003	0.396	4.726	−0.008	0.262
${q}_{0.50}$	6.346	6.274	−0.072	0.396	6.317	−0.029	0.257
${q}_{0.75}$	7.995	7.878	−0.117	0.434	7.961	−0.034	0.270
${q}_{0.95}$	10.336	10.262	−0.074	0.631	10.342	0.006	0.390
	**Average coarseness: 2 days**						
		$\boldsymbol{n}=\textbf{40}$ **(Scenario 7)**			$\boldsymbol{n}=\textbf{100}$ **(Scenario 8)**		
	**True**	**Average**	**Bias**	**RMSE**	**Average**	**Bias**	**RMSE**
Mean	6.403	6.339	−0.064	0.349	6.350	−0.053	0.232
SD	2.327	2.250	−0.077	0.266	2.275	−0.051	0.162
${q}_{0.05}$	2.665	2.766	0.102	0.493	2.712	0.048	0.308
${q}_{0.25}$	4.734	4.735	0.001	0.401	4.722	−0.011	0.254
${q}_{0.50}$	6.346	6.255	−0.091	0.398	6.282	−0.064	0.256
${q}_{0.75}$	7.995	7.831	−0.164	0.447	7.886	−0.109	0.288
${q}_{0.95}$	10.336	10.154	−0.182	0.672	10.202	−0.133	0.434

Abbreviations: RMSE, root mean square error; SD, standard deviation.

^a^ Results are for $S=1000$ simulated datasets and the Weibull incubation density from Backer et al.^8^

**Table 4:** Performance measures for selected features of the incubation density for two levels of data coarseness with $n=40$ and $n=100$.^a^

	**Average coarseness: 1 day**						
		$\boldsymbol{n}=\textbf{40}$ **(Scenario 9)**			$\boldsymbol{n}=\textbf{100}$ **(Scenario 10)**		
	**True**	**Average**	**Bias**	**RMSE**	**Average**	**Bias**	**RMSE**
Mean	7.538	7.535	−0.003	0.726	7.533	−0.005	0.468
SD	4.622	4.565	−0.057	0.237	4.593	−0.029	0.143
${q}_{0.05}$	1.371	1.076	−0.295	0.490	1.229	−0.142	0.293
${q}_{0.25}$	3.050	3.101	0.051	0.604	3.026	−0.024	0.311
${q}_{0.50}$	7.191	7.454	0.263	2.127	7.333	0.142	1.822
${q}_{0.75}$	12.080	11.955	−0.125	0.622	12.027	−0.053	0.315
${q}_{0.95}$	13.734	13.581	−0.153	0.440	13.584	−0.150	0.291
	**Average coarseness: 2 days**						
		$\boldsymbol{n}=\textbf{40}$ **(Scenario 11)**			$\boldsymbol{n}=\textbf{100}$ **(Scenario 12)**		
	**True**	**Average**	**Bias**	**RMSE**	**Average**	**Bias**	**RMSE**
Mean	7.538	7.552	0.014	0.746	7.493	−0.045	0.486
SD	4.622	4.538	−0.084	0.251	4.565	−0.057	0.155
${q}_{0.05}$	1.371	1.141	−0.230	0.468	1.228	−0.143	0.297
${q}_{0.25}$	3.050	3.122	0.072	0.627	3.017	−0.033	0.310
${q}_{0.50}$	7.191	7.508	0.317	2.318	7.275	0.084	1.940
${q}_{0.75}$	12.080	11.972	−0.108	0.517	11.999	−0.081	0.315
${q}_{0.95}$	13.734	13.398	−0.336	0.548	13.376	−0.358	0.445

Abbreviations: RMSE, root mean square error; SD, standard deviation.

^a^ Results are for $S=1000$ simulated datasets and an artificial incubation density constructed as a mixture of two Weibull distributions.

**Table 5:** Performance measures for selected features of the incubation density for two levels of data coarseness with $n=40$ and $n=100$.^a^

	**Average coarseness: 1 day**						
		$\boldsymbol{n}=\textbf{40}$ **(Scenario 13)**			$\boldsymbol{n}=\textbf{100}$ **(Scenario 14)**		
	**True**	**Average**	**Bias**	**RMSE**	**Average**	**Bias**	**RMSE**
Mean	3.810	3.730	−0.080	0.461	3.756	−0.054	0.298
SD	2.889	2.692	−0.197	0.469	2.737	−0.151	0.308
${q}_{0.05}$	0.561	0.582	0.020	0.228	0.564	0.003	0.134
${q}_{0.25}$	1.693	1.731	0.038	0.326	1.721	0.028	0.209
${q}_{0.50}$	3.110	3.128	0.018	0.449	3.135	0.025	0.288
${q}_{0.75}$	5.175	5.075	−0.100	0.659	5.125	−0.050	0.414
${q}_{0.95}$	9.451	8.893	−0.558	1.336	9.063	−0.388	0.867
	**Average coarseness: 2 days**						
		$\boldsymbol{n}=\textbf{40}$ **(Scenario 15)**			$\boldsymbol{n}=\textbf{100}$ **(Scenario 16)**		
	**True**	**Average**	**Bias**	**RMSE**	**Average**	**Bias**	**RMSE**
Mean	3.810	3.519	−0.291	0.492	3.530	−0.280	0.375
SD	2.889	2.411	−0.478	0.603	2.462	−0.426	0.485
${q}_{0.05}$	0.561	0.594	0.032	0.236	0.561	0.000	0.130
${q}_{0.25}$	1.693	1.713	0.020	0.314	1.681	−0.012	0.186
${q}_{0.50}$	3.110	3.020	−0.090	0.426	3.008	−0.102	0.270
${q}_{0.75}$	5.175	4.778	−0.397	0.695	4.820	−0.355	0.503
${q}_{0.95}$	9.451	8.105	−1.346	1.696	8.272	−1.179	1.350

Abbreviations: RMSE, root mean square error; SD, standard deviation.

^a^ Results are for $S=1000$ simulated datasets and the Gamma incubation density from Donnelly et al.^31^

**Table 6:** Proportion of selected models with our methodology across $S=1000$ simulations under different scenarios. First column is the target incubation density.

	$\boldsymbol{n}=\textbf{40}$				$\boldsymbol{n}=\textbf{100}$			
$\sim$ **Lognormal**	**SP**	**LN**	**G**	**W**	**SP**	**LN**	**G**	**W**
(1 day coarseness)	0$\%$	67.7$\%$	25.7$\%$	6.6$\%$	0$\%$	73$\%$	26.5$\%$	0.5$\%$
(2 days coarseness)	0.3$\%$	67$\%$	26.7$\%$	6$\%$	0$\%$	74.3$\%$	23.7$\%$	2$\%$
$\sim$ **Weibull**	$\boldsymbol{n}=\textbf{40}$				$\boldsymbol{n}=\textbf{100}$			
(1 day coarseness)	7.8$\%$	3.4$\%$	19.5$\%$	69.3$\%$	3.9$\%$	0.2$\%$	9.1$\%$	86.8$\%$
(2 days coarseness)	10$\%$	3.1$\%$	17.6$\%$	69.3$\%$	4.5$\%$	0.2$\%$	9.4$\%$	85.9$\%$
$\sim$ **Weibmix**	$\boldsymbol{n}=\textbf{40}$				$\boldsymbol{n}=\textbf{100}$			
(1 day coarseness)	99.8$\%$	0.1$\%$	0$\%$	0.1$\%$	100$\%$	0$\%$	0$\%$	0$\%$
(2 days coarseness)	100$\%$	0$\%$	0$\%$	0$\%$	100$\%$	0$\%$	0$\%$	0$\%$
$\sim$ **Gamma**	$\boldsymbol{n}=\textbf{40}$				$\boldsymbol{n}=\textbf{100}$			
(1 day coarseness)	10.5$\%$	8.9$\%$	45.1$\%$	35.5$\%$	2.7$\%$	1.2$\%$	58.1$\%$	38$\%$
(2 days coarseness)	13.2$\%$	7.6$\%$	38.5$\%$	40.7$\%$	3.9$\%$	0.6$\%$	42.6$\%$	52.9$\%$

Abbreviations: LN, lognormal fit; G, gamma fit; SP, semiparametric fit; W, Weibull fit; Weibmix, mixture of two Weibull distributions.

### Applications to real data

This section applies the proposed flexible estimation methodology to publicly available datasets on reported exposures and symptom onset times. For real analyses, we recommend using at least as many B-spline basis functions as the minimal number used in the simulation study (ie, $K=10$). Here, we use $K=20$, a defensive choice to cope with the eventuality that our data require a very flexible density fit,^16^ and fix $M=\mathrm{20,000}$ for the Markov chain Monte Carlo chain length. A smart choice for ${t}_u$ (and hence ${\tilde{t}}_u$), ie, the upper bound on which to fix the B-spline basis, can for instance be based on information from previous studies on the incubation period for a given pathogen. For instance, Virlogeux et al^32^ report the 99th percentile and range of the incubation period of human avian influenza A (H7N9), and the systematic review of Lessler et al^1^ on incubation periods of acute respiratory viral infections gives an idea of the range of the incubation period for different diseases. Such empirical knowledge can help in finding a choice for ${t}_u$ that supports with high confidence most of the probability mass of the incubation period distribution. Another practical aspect worth mentioning is that exposure times and symptom onset times are in practice reported at a daily time resolution (calendar dates), while our model is in continuous time. A common strategy to transit from discrete to continuous observations is to assume that exact times are uniformly distributed throughout the day and hence to perturb symptom onset times and exposure window bounds by a uniform random variable between $0$ and $1.$^11^Appendix S3 contains arguments regarding the choice of ${t}_u$, detailed datasets (after continuity correction) considered hereafter, as well as estimates for the standard deviation and selected quantiles.

### COVID-19 infections among travelers from Wuhan

First, we attempted to estimate the incubation density based on exposure times and symptom onset dates of confirmed COVID-19 cases with travel history to Wuhan.^8^ The analysis considers 25 visitors to Wuhan with a closed exposure window from which we removed an individual who had a quite large exposure period (20 days) as compared with the remaining observations. Backer et al^8^ obtained a lognormal fit with a mean incubation period of 4.5 days (95% CrI, 3.7-5.6) and a 95th percentile of 8.0 days (95% CrI, 6.3-11.8). From a discussion with the first author of the latter study regarding the analysis of the visitors to Wuhan who had a closed exposure window, we were informed that a Gamma density with a mean of 4.6 days (95% CrI, 3.8-5.4) and a 95th percentile of 7.4 days (95% CrI, 6.2-9.7) fitted equally well. Our methodology provided a similar fit, namely a lognormal density with mean 4.4 days (95% CrI, 4.0-4.8) and a 95th percentile of 7.7 days (95% CrI, 7.2-8.5).

### Transmission pair data on COVID-19

**Figure 6:**
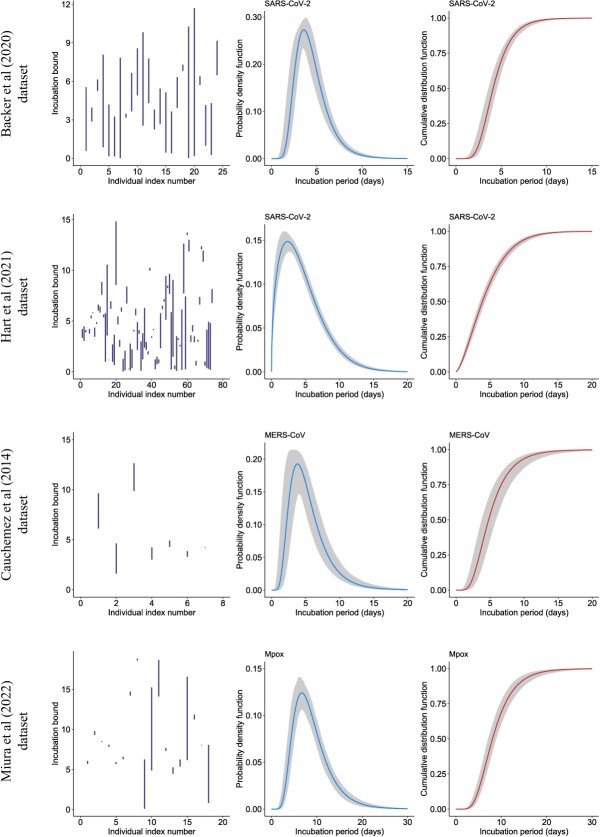
Incubation bounds, estimated probability density function and cumulative distribution function with our flexible Bayesian approach using data on severe acute respiratory syndrome (SARS) coronavirus (CoV)-2,^8^^,^^33^ Middle East respiratory syndrome (MERS)-CoV,^35^ and Mpox.^9^

Next, we considered a dataset on transmission pairs for COVID-19 from Hart et al^33^ that was analyzed in Xia et al.^34^ The latter study obtained a Weibull fit for the incubation density with a mean of 4.9 days (95% CI, 4.4-5.4) and a 95th percentile of 9.9 days (95% CI, 8.9-11.2). Restricting our analysis to a subset of $n=74$ individuals with closed exposure windows that do not exceed 8 days, we obtained a Weibull with a mean of 4.5 days (95% CrI, 4.2-4.9) and a 95th percentile of 10.5 days (95% CrI, 9.8-11.4). Removing the constraint on the exposure window width leads us to a subset of $n=115$ individuals with closed exposure windows (and maximum exposure width of 21.6 days) and we obtained a Weibull fit with mean of 5.5 days (95% CrI, 5.1-5.8) and a 95th percentile of 12.2 days (95% CrI, 11.7-12.9). Figure 6 reports the estimated incubation period with $n=74$.

### Middle East respiratory syndrome

In a third application, we considered a dataset given in Cauchemez et al^35^ that reported lower and upper bounds of the incubation period for 7 individual Middle East respiratory syndrome (MERS) coronavirus (CoV) cases in the United Kingdom, France, Italy, and Tunisia. Based on this data, the latter study obtained a best fit to the incubation density that is lognormal with a mean of 5.5 days (95% CI, 3.6-10.2) and a 95th percentile of 10.2 days, extrapolated from the reported standard deviation in the reference (95% CI, not available). Our approach selects the lognormal fit with a mean of 5.4 days (95% CrI, 4.5-6.5) and a 95th percentile of 10.7 days (95% CrI, 9.5-13.1).

### Mpox

The last application is on a dataset reporting $n=18$ confirmed Mpox cases in the Netherlands.^9^ The latter analysis used a parametric Bayesian approach similar to Backer et al,^8^ and the best fitting model was given by a lognormal distribution with a mean incubation period of 9.0 days (95% CrI, 6.6-10.9) and a 95th percentile of 17.3 days (95% CrI, 13.0-29.0). Analyzing the same dataset with our flexible Bayesian approach, we obtained a lognormal fit with mean incubation period of 8.9 days (95% CrI, 7.9-9.9) and a 95th percentile of 16.6 days (95% CrI, 14.7-19.1).

## Discussion

This article presents a flexible semiparametric approach based on Laplacian-P-splines to tackle the challenging problem of estimating the incubation period distribution based on coarse data. The semiparametric model approximates the incubation density via a finite mixture density smoother, and the latter is used to fit three popular parametric distributions that are often considered in the estimation of incubation times. The Bayesian information criterion is then able to arbitrate between the competing density estimators.

By design, the proposed methodology will perform better than classic parametric models when fitting incubation densities as the latter may not be able to capture incubation distributions characterized by more acute flexibilities. This benefit comes without any overfitting risk thanks to the P-splines smoother and the fact that parametric candidates informed by the semiparametric model are accounted for. At a broader scale, our method can be applied to estimate the incubation period of virtually any infectious disease, provided that intervals of exposure time and symptom onset data are available. Furthermore, it can be used as an intermediate step in mathematical models of infectious diseases (eg, compartmental models) to calibrate the incubation period.

EpiLPS, an R (R Foundation for Statistical Computing, Vienna, Austria) package, provides user-friendly routines to easily estimate the incubation distribution based on the flexible method described here. This can be done at a relatively low computational cost thanks to integration of C++ code for some subroutines in EpiLPS via the Rcpp package.^36^ Further documentation and examples are also available on the associated website (https://epilps.com/). Simulation results and real data applications in this paper can be reproduced by using the code available at the GitHub repository linked in the Data Availability statement below.

From here, several interesting research paths can be explored. The present model can for instance be enriched by not only considering a two-component mixture in the semiparametric approach, but also a multiple-component mixture with a multiple imputation approach. Another possibility is to extend our model to handle estimation of the generation interval (time difference between infection events of a primary case and a secondary case) by working under a convolution setting. We also noticed that our approach consistently produces narrower credible intervals as compared with other studies. It would be thus interesting to compare the coverage performance of credible intervals obtained with our flexible approach against more traditional parametric models.

## Supplementary Material

Web_Material_kwae192

## Data Availability

Simulation results and real data applications in this paper can be fully reproduced with the code available on the GitHub repository https://github.com/oswaldogressani/Incubation based on the EpiLPS package version 1.3.0. EpiLPS is an R (R Foundation for Statistical Computing, Vienna, Austria) package available at https://cran.r-project.org/package=EpiLPS.
