# Supplementary material for: Flexible Bayesian estimation of incubation times
Source: Am J Epidemiol. 2024 Jul 10;194(2):490–501. doi: 10.1093/aje/kwae192 (PMC11815507; doi:10.1093/aje/kwae192)
Supplement: Web_Material_kwae192 [file web_material_kwae192.pdf]

Supplementary Material: Flexible Bayesian estimation of incubation times

Oswaldo Gressani, Andrea Torneri, Niel Hens, Christel Faes

Table of contents

Appendix S1 . . . . **2**

Appendix S2 . . . . **6**

Appendix S3 and Tables S1-S13. . . . .**9**

References **20**

Appendix S1

The Laplace approximation

The Laplace approximation to the conditional posterior of the B-spline amplitudes is simply a Gaussian density fitted around the (unknown) mode $\boldsymbol{\theta}_M(\lambda)$ of $p(\boldsymbol{\theta}|\lambda, \mathcal{D})$. The modal value $\boldsymbol{\theta}_M(\lambda)$ is approached via a Newton-Raphson algorithm involving the gradient and Hessian matrix of $\log p(\boldsymbol{\theta}|\lambda, \mathcal{D})$, so that at convergence, one recovers the Laplace approximation $\widetilde{p}_G(\boldsymbol{\theta}|\lambda, \mathcal{D})$ with mean/mode $\boldsymbol{\theta}^*(\lambda) \approx \boldsymbol{\theta}_M(\lambda)$ and variance-covariance matrix equal to the inverse of the negative Hessian matrix of $\log p(\boldsymbol{\theta}|\lambda, \mathcal{D})$ evaluated at $\boldsymbol{\theta}^*(\lambda)$ denoted by $\Sigma^*(\lambda)$. To speed-up the mode finding algorithm, we compute the following analytical versions of the gradient and Hessian:

$$\nabla_{\boldsymbol{\theta}} \log p(\boldsymbol{\theta}|\lambda, \mathcal{D}) = \nabla_{\boldsymbol{\theta}} \ell(\boldsymbol{\theta}; \mathcal{D}) - \lambda P \boldsymbol{\theta},$$

$$\nabla^2_{\boldsymbol{\theta}} \log p(\boldsymbol{\theta}|\lambda, \mathcal{D}) = \nabla^2_{\boldsymbol{\theta}} \ell(\boldsymbol{\theta}; \mathcal{D}) - \lambda P.$$

To ease the notation, we define the following quantities related to the left bound of the incubation period $\psi^L_{ik} := \sum_{j=1}^{j(t_i^{\mathcal{I}_L})} h(s_j) b_k(s_j)\Delta$, $\psi^L_{il} := \sum_{j=1}^{j(t_i^{\mathcal{I}_L})} h(s_j) b_l(s_j)\Delta$, $\psi^L_{ikl} := \sum_{j=1}^{j(t_i^{\mathcal{I}_L})} h(s_j) b_k(s_j) b_l(s_j)\Delta$ and analogously for the right bound ψ^R_{ik}, ψ^R_{il} and ψ^R_{ikl}. The gradient of the log-likelihood is:

$$\begin{aligned}
\nabla_{\boldsymbol{\theta}} \ell(\boldsymbol{\theta}; \mathcal{D}) &= \left(\frac{\partial}{\partial \theta_1}\ell(\boldsymbol{\theta}; \mathcal{D}), \ldots, \frac{\partial}{\partial \theta_K}\ell(\boldsymbol{\theta}; \mathcal{D})\right)^\top, \text{where} \\
\frac{\partial}{\partial \theta_k}\ell(\boldsymbol{\theta}; \mathcal{D}) &= \sum_{i=1}^n \left(\widetilde{S}(t_i^{\mathcal{I}_L}) - \widetilde{S}(t_i^{\mathcal{I}_R})\right)^{-1}\left(\widetilde{S}(t_i^{\mathcal{I}_R})\psi^R_{ik} - \widetilde{S}(t_i^{\mathcal{I}_L})\psi^L_{ik}\right), \text{ for } k = 1, \ldots, K.
\end{aligned}$$

The Hessian matrix of the log-likelihood is:

$$\begin{aligned}
\nabla^2_{\boldsymbol{\theta}} \ell(\boldsymbol{\theta}; \mathcal{D}) &= \frac{\partial^2}{\partial \boldsymbol{\theta} \partial \boldsymbol{\theta}^\top}\ell(\boldsymbol{\theta}; \mathcal{D}), \text{ where the entry at the } k\text{th row and } l\text{th column is:} \\
\frac{\partial^2}{\partial \theta_k \partial \theta_l}\ell(\boldsymbol{\theta}; \mathcal{D}) &= \sum_{i=1}^n \Bigg\{ \left(\widetilde{S}(t_i^{\mathcal{I}_L}) - \widetilde{S}(t_i^{\mathcal{I}_R})\right)^{-1}\left\{\widetilde{S}(t_i^{\mathcal{I}_R})\left(\psi^R_{ikl} - \psi^R_{il}\psi^R_{ik}\right) - \widetilde{S}(t_i^{\mathcal{I}_L})\left(\psi^L_{ikl} - \psi^L_{il}\psi^L_{ik}\right)\right\} \\
&\quad - \left(\widetilde{S}(t_i^{\mathcal{I}_R})\psi^R_{ik} - \widetilde{S}(t_i^{\mathcal{I}_L})\psi^L_{ik}\right)\left(\widetilde{S}(t_i^{\mathcal{I}_R})\psi^R_{il} - \widetilde{S}(t_i^{\mathcal{I}_L})\psi^L_{il}\right)\left(\widetilde{S}(t_i^{\mathcal{I}_L}) - \widetilde{S}(t_i^{\mathcal{I}_R})\right)^{-2}\Bigg\}.
\end{aligned}$$

Using the above gradient and Hessian, an iterative algorithm (e.g. Newton-Raphson) can be used to obtain $\boldsymbol{\theta}^*(\lambda)$ as a proxy for the posterior mode of $p(\boldsymbol{\theta}|\lambda, \mathcal{D})$. The mode of the Laplace approximation is conditional on the penalty parameter and we therefore need a strategy to calibrate the amount of smoothing. The idea is to use an optimal smoothing approach where the maximum *a posteriori* value of an approximate version of the marginal posterior of λ is computed (1, 2). Mathematically, optimal smoothing means $\lambda_M = \text{argmax}_\lambda \log \widetilde{p}(\lambda|\mathcal{D})$, with the following (approximate) posterior distribution for the penalty:

$$\begin{aligned}
\widetilde{p}(\lambda|\mathcal{D}) &\propto \left.\frac{\mathcal{L}(\boldsymbol{\theta}; \mathcal{D}) p(\boldsymbol{\theta}|\lambda) p(\lambda)}{\widetilde{p}_G(\boldsymbol{\theta}|\lambda, \mathcal{D})}\right|_{\boldsymbol{\theta}=\boldsymbol{\theta}^*(\lambda)} \\
&\propto |\Sigma^*(\lambda)|^{0.5} \lambda^{0.5K + a_\lambda - 1} \exp\left(\ell(\boldsymbol{\theta}^*(\lambda); \mathcal{D}) - \lambda(0.5\boldsymbol{\theta}^{*\top}(\lambda) P \boldsymbol{\theta}^*(\lambda) + b_\lambda)\right).
\end{aligned}$$

An approximation to λ_M denoted by λ^* is found by exploring $\log \widetilde{p}(\lambda|\mathcal{D})$ on a linear grid for $\log_{10}(\lambda)$ and the final resulting Laplace approximation is written as $\widetilde{p}_G(\boldsymbol{\theta}|\lambda^*, \mathcal{D}) = \mathcal{N}_{\dim(\boldsymbol{\theta})}\left(\boldsymbol{\theta}^*(\lambda^*), \Sigma^*(\lambda^*)\right)$.

Detailed derivations

Gradient

Recall that the (approximated) log-likelihood is:

$$\begin{aligned}
\ell(\boldsymbol{\theta};\mathcal{D}) &= \sum_{i=1}^{n}\log\left(\exp\left(-\sum_{j=1}^{j(t_i^{\mathcal{I}_L})}\exp(\boldsymbol{\theta}^\top b(s_j))\Delta\right)-\exp\left(-\sum_{j=1}^{j(t_i^{\mathcal{I}_R})}\exp(\boldsymbol{\theta}^\top b(s_j))\Delta\right)\right)\\
&= \sum_{i=1}^{n}\log\left(\widetilde{S}(t_i^{\mathcal{I}_L})-\widetilde{S}(t_i^{\mathcal{I}_R})\right).\\
\frac{\partial}{\partial\theta_k}\ell(\boldsymbol{\theta};\mathcal{D}) &= \sum_{i=1}^{n}\left(\widetilde{S}(t_i^{\mathcal{I}_L})-\widetilde{S}(t_i^{\mathcal{I}_R})\right)^{-1}\left(\frac{\partial}{\partial\theta_k}\widetilde{S}(t_i^{\mathcal{I}_L})-\frac{\partial}{\partial\theta_k}\widetilde{S}(t_i^{\mathcal{I}_R})\right).
\end{aligned}$$

Note that:

$$\begin{aligned}
\frac{\partial}{\partial\theta_k}\widetilde{S}(t_i^{\mathcal{I}_L}) &= -\exp\left(-\sum_{j=1}^{j(t_i^{\mathcal{I}_L})}h(s_j)\Delta\right)\sum_{j=1}^{j(t_i^{\mathcal{I}_L})}h(s_j)b_k(s_j)\Delta\\
&= -\widetilde{S}(t_i^{\mathcal{I}_L})\psi_{ik}^{L}.\\
\frac{\partial}{\partial\theta_k}\widetilde{S}(t_i^{\mathcal{I}_R}) &= -\widetilde{S}(t_i^{\mathcal{I}_R})\psi_{ik}^{R}.
\end{aligned}$$

It follows that the kth entry to $\nabla_{\boldsymbol{\theta}}\ell(\boldsymbol{\theta};\mathcal{D})$ is:

$$\frac{\partial}{\partial\theta_k}\ell(\boldsymbol{\theta};\mathcal{D}) = \sum_{i=1}^{n}\left(\widetilde{S}(t_i^{\mathcal{I}_L})-\widetilde{S}(t_i^{\mathcal{I}_R})\right)^{-1}\left(\widetilde{S}(t_i^{\mathcal{I}_R})\psi_{ik}^{R}-\widetilde{S}(t_i^{\mathcal{I}_L})\psi_{ik}^{L}\right).$$

Hessian

Let us define:

$$\begin{aligned}
\gamma_i(\boldsymbol{\theta}) &:= \widetilde{S}(t_i^{\mathcal{I}_R})\psi_{ik}^{R}-\widetilde{S}(t_i^{\mathcal{I}_L})\psi_{ik}^{L},\\
\eta_i(\boldsymbol{\theta}) &:= \widetilde{S}(t_i^{\mathcal{I}_L})-\widetilde{S}(t_i^{\mathcal{I}_R}),
\end{aligned}$$

so that the kth entry to $\nabla_{\boldsymbol{\theta}}\ell(\boldsymbol{\theta};\mathcal{D})$ is rewritten compactly as:

$$\frac{\partial}{\partial\theta_k}\ell(\boldsymbol{\theta};\mathcal{D}) = \sum_{i=1}^{n}\frac{\gamma_i(\boldsymbol{\theta})}{\eta_i(\boldsymbol{\theta})}.$$

Deriving the above expression again with respect to the lth B-spline component gives:

$$\begin{aligned}
\frac{\partial^2}{\partial\theta_k\partial\theta_l}\ell(\boldsymbol{\theta};\mathcal{D}) &= \sum_{i=1}^{n}\Big(\eta_i(\boldsymbol{\theta})\Big)^{-2}\left(\frac{\partial\gamma_i(\boldsymbol{\theta})}{\partial\theta_l}\eta_i(\boldsymbol{\theta})-\gamma_i(\boldsymbol{\theta})\frac{\partial\eta_i(\boldsymbol{\theta})}{\partial\theta_l}\right)\\
&= \sum_{i=1}^{n}\Big(\eta_i(\boldsymbol{\theta})\Big)^{-1}\frac{\partial\gamma_i(\boldsymbol{\theta})}{\partial\theta_l}-\gamma_i(\boldsymbol{\theta})\frac{\partial\eta_i(\boldsymbol{\theta})}{\partial\theta_l}\Big(\eta_i(\boldsymbol{\theta})\Big)^{-2}.
\end{aligned}$$

$$
\begin{aligned}
\frac{\partial \gamma_i(\boldsymbol{\theta})}{\partial \theta_l} &= \frac{\partial}{\partial \theta_l}\left(\widetilde{S}(t_i^{\mathcal{I}_R})\psi_{ik}^R - \widetilde{S}(t_i^{\mathcal{I}_L})\psi_{ik}^L\right) \\
&= \left(\frac{\partial \widetilde{S}(t_i^{\mathcal{I}_R})}{\partial \theta_l}\psi_{ik}^R + \widetilde{S}(t_i^{\mathcal{I}_R})\frac{\partial \psi_{ik}^R}{\partial \theta_l}\right) - \left(\frac{\partial \widetilde{S}(t_i^{\mathcal{I}_L})}{\partial \theta_l}\psi_{ik}^L + \widetilde{S}(t_i^{\mathcal{I}_L})\frac{\partial \psi_{ik}^L}{\partial \theta_l}\right) \\
&= \left(-\widetilde{S}(t_i^{\mathcal{I}_R})\psi_{il}^R\psi_{ik}^R + \widetilde{S}(t_i^{\mathcal{I}_R})\psi_{ikl}^R\right) - \left(-\widetilde{S}(t_i^{\mathcal{I}_L})\psi_{il}^L\psi_{ik}^L + \widetilde{S}(t_i^{\mathcal{I}_L})\psi_{ikl}^L\right) \\
&= \widetilde{S}(t_i^{\mathcal{I}_R})\left(\psi_{ikl}^R - \psi_{il}^R\psi_{ik}^R\right) - \widetilde{S}(t_i^{\mathcal{I}_L})\left(\psi_{ikl}^L - \psi_{il}^L\psi_{ik}^L\right).
\end{aligned}
$$

$$
\begin{aligned}
\frac{\partial \eta_i(\boldsymbol{\theta})}{\partial \theta_l} &= \frac{\partial}{\partial \theta_l}\left(\widetilde{S}(t_i^{\mathcal{I}_L}) - \widetilde{S}(t_i^{\mathcal{I}_R})\right) \\
&= \frac{\partial \widetilde{S}(t_i^{\mathcal{I}_L})}{\partial \theta_l} - \frac{\partial \widetilde{S}(t_i^{\mathcal{I}_R})}{\partial \theta_l} \\
&= -\widetilde{S}(t_i^{\mathcal{I}_L})\psi_{il}^L - \left(-\widetilde{S}(t_i^{\mathcal{I}_R})\psi_{il}^R\right) \\
&= \widetilde{S}(t_i^{\mathcal{I}_R})\psi_{il}^R - \widetilde{S}(t_i^{\mathcal{I}_L})\psi_{il}^L.
\end{aligned}
$$

$$
\begin{aligned}
\frac{\partial^2}{\partial \theta_k \partial \theta_l}\ell(\boldsymbol{\theta}; \mathcal{D}) &= \sum_{i=1}^{n}\Bigg\{\big(\widetilde{S}(t_i^{\mathcal{I}_L}) - \widetilde{S}(t_i^{\mathcal{I}_R})\big)^{-1}\Big\{\widetilde{S}(t_i^{\mathcal{I}_R})\big(\psi_{ikl}^R - \psi_{il}^R\psi_{ik}^R\big) - \widetilde{S}(t_i^{\mathcal{I}_L})\big(\psi_{ikl}^L - \psi_{il}^L\psi_{ik}^L\big)\Big\} \\
&\quad -\Big(\widetilde{S}(t_i^{\mathcal{I}_R})\psi_{ik}^R - \widetilde{S}(t_i^{\mathcal{I}_L})\psi_{ik}^L\Big)\Big(\widetilde{S}(t_i^{\mathcal{I}_R})\psi_{il}^R - \widetilde{S}(t_i^{\mathcal{I}_L})\psi_{il}^L\Big)\big(\widetilde{S}(t_i^{\mathcal{I}_L}) - \widetilde{S}(t_i^{\mathcal{I}_R})\big)^{-2}\Bigg\}.
\end{aligned}
$$

Appendix S2

Moment matching approach to fit parametric families

This Web Appendix aims at explaining how the moment matching approach is used to fit the two-parameter lognormal, Gamma and Weibull distributions based on our semi-parametric model. We use α and β to generically denote the two parameters of the latter families (see below for the detailed parameterization). The moment matching strategy is as follows:

Moment matching algorithm to fit parametric distributions.

1: **for** $m = 1, \ldots, M$ **do**

2: From the LGS MCMC sample, compute $\widehat{\varphi}_{SP}(t|\boldsymbol{\theta}^{(m)}) = 0.5\Big(\widehat{\varphi}_{IC}(t|\boldsymbol{\theta}^{(m)}) + \widehat{\varphi}_{HS}(t)\Big)$.

3: Obtain (numerically) the first moment and second central moment of $\mathcal{I}$ as:

$$\begin{aligned}\widehat{E}^{(m)}(\mathcal{I}) &= \int_0^{+\infty} t\ \widehat{\varphi}_{SP}(t|\boldsymbol{\theta}^{(m)})\ dt,\\ \widehat{V}^{(m)}(\mathcal{I}) &= \int_0^{+\infty} (t-\widehat{E}^{(m)}(\mathcal{I}))^2\ \widehat{\varphi}_{SP}(t|\boldsymbol{\theta}^{(m)})\ dt.\end{aligned}$$

4: Use the above moments to estimate $\alpha^{(m)}$ and $\beta^{(m)}$ for the chosen parametric distribution.

5: **end for**

The posterior median of the samples $\{\alpha^{(m)} : m = 1, \ldots, M\}$ and $\{\beta^{(m)} : m = 1, \ldots, M\}$ denoted by $\widehat{\alpha}$ and $\widehat{\beta}$, respectively, can be used to construct the lognormal density fit $\widehat{\varphi}_{LN}(t)$, the Gamma density fit $\widehat{\varphi}_G(t)$ and the Weibull density fit $\widehat{\varphi}_W(t)$ to $\varphi(t)$. To choose between the four candidate density estimates $\{\widehat{\varphi}_{SP}(\cdot), \widehat{\varphi}_{LN}(\cdot), \widehat{\varphi}_G(\cdot), \widehat{\varphi}_W(\cdot)\}$, we use the Bayesian information criterion (3) computed as $\text{BIC}_{\mathcal{P}} = -2\ell(\widehat{\alpha}, \widehat{\beta}; \mathcal{D}) + 2\log(n)$ for the parametric fits, i.e. $\mathcal{P} \in \{LN, G, W\}$ and $\ell(\widehat{\alpha}, \widehat{\beta}; \mathcal{D}) = \sum_{i=1}^n \log\big(\int_{t_i^{\mathcal{I}_L}}^{t_i^{\mathcal{I}_R}} \widehat{\varphi}_{\mathcal{P}}(t)dt\big)$. For the semi-parametric fit with P-splines, we use the formula $\text{BIC}_{SP} = -2\ell(\widehat{\boldsymbol{\theta}}; \mathcal{D}) + \text{ED}\log(n)$, where $\widehat{\boldsymbol{\theta}}$ is the estimate of $\boldsymbol{\theta}$ obtained from the LGS algorithm and ED is the effective dimension of the model obtained as follows $\text{ED} = \text{Tr}\big(\big(-\nabla^2_{\boldsymbol{\theta}}\ell(\widehat{\boldsymbol{\theta}}; \mathcal{D}) + \widehat{\lambda}P\big)^{-1}\big(-\nabla^2_{\boldsymbol{\theta}}\ell(\widehat{\boldsymbol{\theta}}; \mathcal{D})\big)\big)$, where $\widehat{\lambda}$ is the median of the MCMC sample for λ in the LGS algorithm and $\text{Tr}(\cdot)$ denotes the trace of a matrix.

The simplicity of the latter formula and its straightforward evaluation is the main rationale for choosing the BIC here. Although leave-one-out cross-validation or the widely applicable information criterion (WAIC) provide a more refined basis for model selection, they are less appealing due to implementation complexity and larger computational burden (4).

Parameterization of the Lognormal, Gamma and Weibull

**Lognormal distribution**	
Notation	$X \sim LogNorm(\alpha, \beta^2)$
Parameters	$\alpha \in \mathbb{R}$ location; $\beta > 0$ scale
Density function	$p(x) = \frac{1}{x\sqrt{2\pi\beta^2}} \exp\Big(-\frac{1}{2}\Big(\frac{\log(x)-\alpha}{\beta}\Big)^2\Big)$
Support	$x > 0$
1st moment (Mean)	$E(X) = \exp\Big(\alpha + \frac{\beta^2}{2}\Big)$
2nd central moment (Variance)	$V(X) = \exp\big(2\alpha + \beta^2\big)\big(\exp\big(\beta^2\big) - 1\big)$
Moment matching	Root finding algorithm
**Gamma distribution**	
Notation	$X \sim \mathcal{G}(\alpha, \beta)$
Parameters	$\alpha > 0$ shape; $\beta > 0$ rate
Density function	$p(x) = \frac{\beta^\alpha}{\Gamma(a)} x^{\alpha-1} \exp\big(-\beta x\big)$
Support	$x > 0$
1st moment (Mean)	$E(X) = \frac{\alpha}{\beta}$
2nd central moment (Variance)	$V(X) = \frac{\alpha}{\beta^2}$
Moment matching	Analytically available
**Weibull distribution**	
Notation	$X \sim Weibull(\alpha, \beta)$
Parameters	$\alpha > 0$ shape; $\beta > 0$ scale
Density function	$p(x) = \frac{\alpha}{\beta^\alpha} x^{\alpha-1} \exp\Big(-\Big(\frac{x}{\beta}\Big)^\alpha\Big)$
Support	$x > 0$
1st moment (Mean)	$E(X) = \beta\Gamma(1 + \frac{1}{\alpha})$
2nd central moment (Variance)	$V(X) = \beta^2\Big(\Gamma(1 + \frac{2}{\alpha}) - \big(\Gamma(1 + \frac{1}{\alpha})\big)^2\Big)$
Moment matching	Root finding algorithm

Description of the parametric distributions used in the moment matching approach.

Appendix S3

Simulation study with a large sample size (n = 200)

Assuming the same target incubation densities as in the main manuscript, we implement 8 additional simulation scenarios to assess how our method performs in large samples with n=200. Tables S1-S4 below summarize the results for selected pointwise features of the incubation density.

**Lognormal (n=200)**		Average coarseness: 1 day (Scenario 1)			Average coarseness: 2 days (Scenario 2)		
	True	Average	Bias	RMSE	Average	Bias	RMSE
Mean	5.528	5.472	-0.056	0.158	5.417	-0.111	0.186
SD	2.075	1.997	-0.078	0.154	1.934	-0.141	0.191
$q_{0.05}$	2.849	2.837	-0.012	0.131	2.847	-0.002	0.134
$q_{0.25}$	4.052	4.047	-0.005	0.124	4.036	-0.016	0.127
$q_{0.50}$	5.176	5.156	-0.020	0.147	5.118	-0.057	0.158
$q_{0.75}$	6.612	6.546	-0.066	0.203	6.467	-0.145	0.241
$q_{0.95}$	9.403	9.181	-0.222	0.426	9.002	-0.401	0.536

Table S1. Performance measures for selected features of the incubation density for two levels of data coarseness with sample size n=200. Results are for S=1000 simulated datasets and the lognormal incubation density of (5).

**Weibull (n=200)**		Average coarseness: 1 day (Scenario 3)			Average coarseness: 2 days (Scenario 4)		
	True	Average	Bias	RMSE	Average	Bias	RMSE
Mean	6.403	6.392	-0.011	0.169	6.338	-0.065	0.181
SD	2.327	2.327	0.001	0.110	2.282	-0.045	0.116
$q_{0.05}$	2.665	2.666	0.002	0.208	2.666	0.002	0.202
$q_{0.25}$	4.734	4.722	-0.012	0.191	4.705	-0.029	0.190
$q_{0.50}$	6.346	6.327	-0.019	0.188	6.282	-0.064	0.198
$q_{0.75}$	7.995	7.975	-0.020	0.199	7.893	-0.102	0.225
$q_{0.95}$	10.336	10.334	-0.002	0.285	10.187	-0.149	0.321

Table S2. Performance measures for selected features of the incubation density for two levels of data coarseness with sample size n=200. Results are for S=1000 simulated datasets and the Weibull incubation density of (6).

**Weibull mixture (n=200)**		Average coarseness: 1 day (Scenario 5)			Average coarseness: 2 days (Scenario 6)		
	True	Average	Bias	RMSE	Average	Bias	RMSE
Mean	7.538	7.532	-0.006	0.327	7.506	-0.032	0.328
SD	4.622	4.599	-0.023	0.098	4.578	-0.044	0.110
$q_{0.05}$	1.371	1.280	-0.091	0.197	1.282	-0.089	0.196
$q_{0.25}$	3.050	3.005	-0.045	0.207	2.996	-0.054	0.210
$q_{0.50}$	7.191	7.238	0.092	1.561	7.248	0.057	1.627
$q_{0.75}$	12.080	12.023	-0.057	0.215	12.005	-0.075	0.219
$q_{0.95}$	13.734	13.594	-0.140	0.225	13.407	-0.327	0.377

Table S3. Performance measures for selected features of the incubation density for two levels of data coarseness with sample size n=200. Results are for S=1000 simulated datasets and an artificial bimodal incubation density constructed as a mixture of two Weibull distributions.

**Gamma (*n*=200)**		Average coarseness: 1 day (Scenario 7)			Average coarseness: 2 days (Scenario 8)		
	True	Average	Bias	RMSE	Average	Bias	RMSE
Mean	3.810	3.738	-0.072	0.205	3.528	-0.282	0.333
SD	2.889	2.745	-0.144	0.237	2.472	-0.417	0.449
$q_{0.05}$	0.561	0.554	-0.008	0.091	0.560	-0.002	0.089
$q_{0.25}$	1.693	1.699	0.006	0.136	1.675	-0.018	0.131
$q_{0.50}$	3.110	3.109	-0.001	0.189	3.001	-0.109	0.210
$q_{0.75}$	5.175	5.105	-0.070	0.282	4.818	-0.357	0.438
$q_{0.95}$	9.451	9.073	-0.377	0.649	8.300	-1.151	1.244

Table S4. Performance measures for selected features of the incubation density for two levels of data coarseness with sample size *n*=200. Results are for *S*=1000 simulated datasets and the Gamma incubation density of (7).

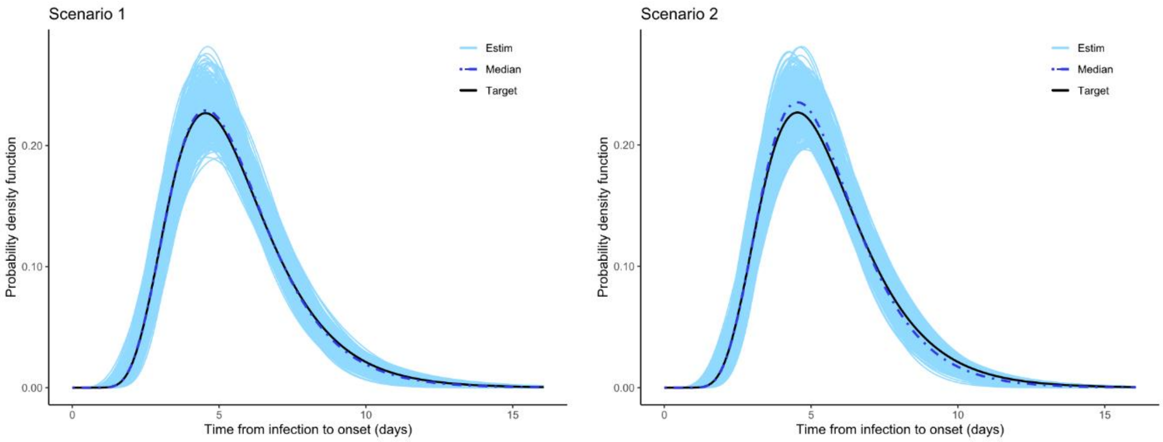

Figure S1. Estimated incubation densities (blue) when the target incubation density (black) is lognormal and the sample size is *n*=200. The dash-dotted line is the pointwise median across the *S*=1000 simulated datasets. Left panel is for an average data coarseness of 1 day (Scenario 1) and right panel for an average coarseness of 2 days (Scenario 2).

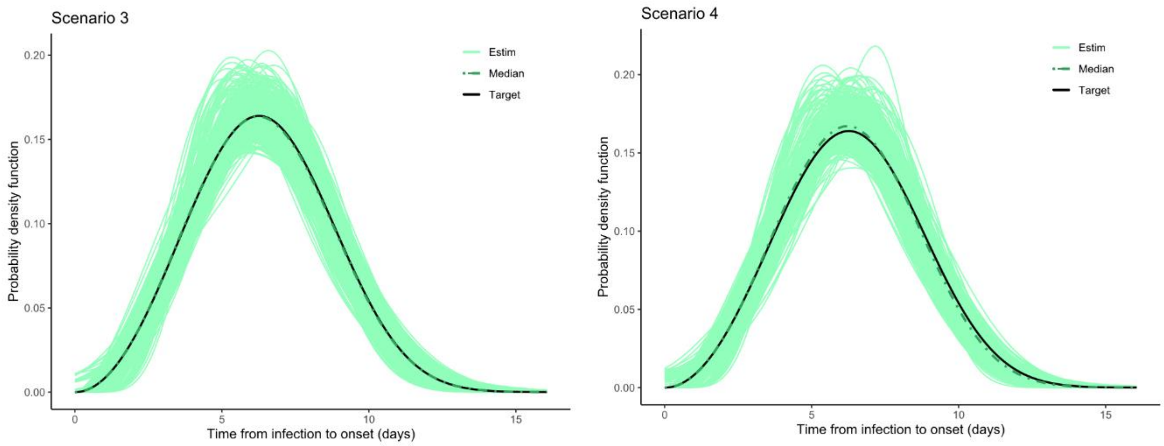

Figure S2. Estimated incubation densities (green) when the target incubation density (black) is Weibull and the sample size is *n*=200. The dash-dotted line is the pointwise median across the *S*=1000 simulated datasets. Left panel is for an average data coarseness of 1 day (Scenario 3) and right panel for an average coarseness of 2 days (Scenario 4).

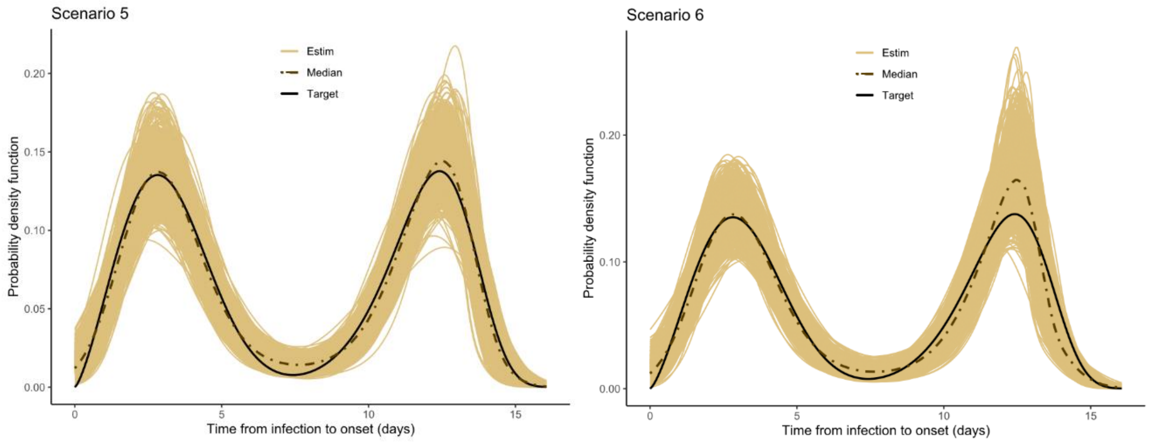

Figure S3. Estimated incubation densities (brown) when the target incubation density (black) is a mixture of two Weibull distributions and the sample size is n=200. The dash-dotted line is the pointwise median across the S=1000 simulated datasets. Left panel is for an average data coarseness of 1 day (Scenario 5) and right panel for an average coarseness of 2 days (Scenario 6).

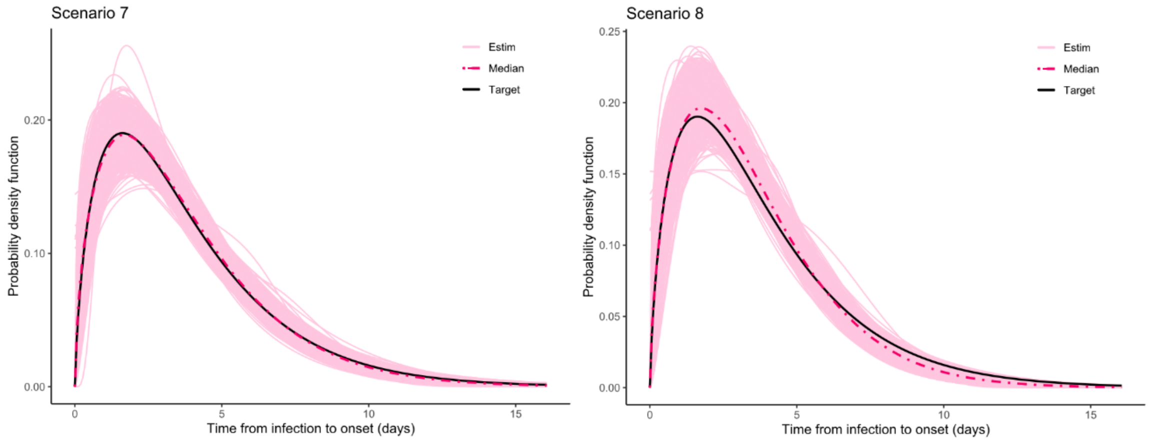

Figure S4. Estimated incubation densities (pink) when the target incubation density (black) is Gamma and the sample size is n=200. The dash-dotted line is the pointwise median across the S=1000 simulated datasets. Left panel is for an average data coarseness of 1 day (Scenario 7) and right panel for an average coarseness of 2 days (Scenario 8).

	Average coarseness: 1 day				Average coarseness: 2 days			
Target	SP	LN	G	W	SP	LN	G	W
**Lognormal**	0%	81.6%	18.4%	0%	0%	79.4%	20.6%	0%
**Weibull**	1.8%	0%	3.2%	95%	3.6%	0%	2.6%	93.8%
**Weibull mixture**	100%	0%	0%	0%	100%	0%	0%	0%
**Gamma**	0.5%	0.1%	62.8%	36.6%	0.5%	0%	42.8%	56.7%

Table S5. Proportion of selected models across S=1000 simulations under different scenarios. The target incubation distribution is in the first column. Remaining columns show the proportion of selected models where SP is the semi-parametric model, LN is the lognormal model, G is the Gamma model and W is the Weibull model. Results are for n=200 and two levels of data coarseness.

Sensitivity analyses

We assess the sensitivity of our methodology to the choice of K (the number of B-spline basis functions) and to the choice of the shape and rate parameters (a_λ and b_λ, respectively) for the Gamma prior imposed on the penalty parameter λ. For the data generating mechanism, the lognormal incubation density (see main manuscript) of (5) is chosen with a sample size of n=100 and an average data coarseness of 1 day. Results are summarized in Tables S6-S7.

From Table S6, we see that the estimates of selected features of the incubation density are not very sensitive to the choice of K as translated by the average of the estimates and the RMSE. The latter quantities are almost equal for K=12, K=15 and K=20. A similar conclusion is reached based on results from Table S7, namely that there is little sensitivity of our methodology to the choice of the shape and rate parameters in the Gamma prior imposed on the penalty parameter.

Lognormal		K=12			K=15			K=20		
	True	Average	Bias	RMSE	Average	Bias	RMSE	Average	Bias	RMSE
Mean	5.528	5.447	-0.052	0.214	5.513	-0.016	0.216	5.507	-0.021	0.211
SD	2.075	1.995	-0.080	0.201	2.029	-0.046	0.215	2.020	-0.055	0.212
$q_{0.05}$	2.849	2.824	-0.025	0.175	2.824	-0.025	0.182	2.827	-0.022	0.178
$q_{0.25}$	4.052	4.052	0.000	0.175	4.064	0.013	0.174	4.065	0.013	0.172
$q_{0.50}$	5.176	5.169	-0.007	0.206	5.197	0.021	0.205	5.193	0.017	0.202
$q_{0.75}$	6.612	6.561	-0.051	0.275	6.609	-0.003	0.281	6.600	-0.012	0.276
$q_{0.95}$	9.403	9.173	-0.230	0.557	9.278	-0.126	0.595	9.253	-0.151	0.581

Table S6. Sensitivity of selected features of the incubation density to the number K of B-spline basis functions across S=1000 simulations. The target incubation distribution is the lognormal density reported in (5). Results are for n=100 and an average data coarseness of 1 day.

Lognormal		$a_\lambda = b_\lambda = 10^{-3}$			$a_\lambda = b_\lambda = 10^{-2}$			$a_\lambda = b_\lambda = 10^{-1}$		
	True	Average	Bias	RMSE	Average	Bias	RMSE	Average	Bias	RMSE
Mean	5.528	5.464	-0.065	0.212	5.469	-0.059	0.204	5.475	-0.053	0.202
SD	2.075	1.976	-0.098	0.207	1.984	-0.091	0.206	1.986	-0.088	0.211
$q_{0.05}$	2.849	2.832	-0.017	0.171	2.827	-0.022	0.172	2.831	-0.018	0.180
$q_{0.25}$	4.052	4.052	0.000	0.169	4.052	0.000	0.165	4.056	0.005	0.171
$q_{0.50}$	5.176	5.161	-0.015	0.200	5.165	-0.011	0.193	5.170	-0.005	0.194
$q_{0.75}$	6.612	6.539	-0.073	0.273	6.549	-0.063	0.265	6.556	-0.056	0.261
$q_{0.95}$	9.403	9.123	-0.280	0.575	9.143	-0.261	0.565	9.154	-0.250	0.566

Table S7. Sensitivity of selected features of the incubation density to the shape parameter a_λ and rate parameter b_λ of the Gamma prior imposed on the penalty parameter λ across S=1000 simulations. The target incubation distribution is the lognormal density reported in (5). Results are for n=100, K=10 and an average data coarseness of 1 day.

Dataset on COVID-19 infections among travellers from Wuhan

The dataset was obtained from the Supplementary Material of (6) (https://doi.org/10.2807/1560-7917.ES.2020.25.5.2000062). For this dataset, the largest observed right bound of the incubation period (after continuity correction) is given by 11.707 days (see last column of Table S8 below). The parametric distributions (Weibull, Gamma and lognormal) fitted in (6) for the incubation period reveal an estimated 99th percentile of 11.9 days for the Weibull, 14.1 days for the Gamma and 18.5 days for the lognormal. In addition, point estimates of the 95th percentile of estimated incubation periods for coronaviruses from different studies given in Table 3 of the latter reference do not exceed 19.7 days. This empirical knowledge motivates our choice of t_u=20 for the upper bound of the incubation period so that the B-spline basis is specified on the compact domain [0, 20]. This conservative range supports with high confidence most of the probability mass of the true underlying incubation period distribution. Values in Table S8 can be reproduced from: https://github.com/oswaldogressani/Incubation/.

i	$t_i^{E_L}$	$t_i^{E_R}$	$\mathcal{E}_i$	t_i^S	$t_i^{\mathcal{I}_L}$	$t_i^{\mathcal{I}_R}$
1	9.286	14.267	4.981	14.837	0.570	5.551
2	21.232	22.317	1.084	25.187	2.870	3.954
3	14.159	15.040	0.881	20.303	5.263	6.144
4	19.831	27.046	7.214	27.915	0.869	8.083
5	23.265	27.305	4.039	27.456	0.151	4.191
6	24.553	27.646	3.093	27.807	0.161	3.254
7	19.677	27.485	7.808	27.502	0.017	7.825
8	21.765	22.074	0.308	25.244	3.170	3.478
9	23.504	26.494	2.990	30.153	3.659	6.649
10	22.175	25.848	3.674	30.751	4.903	8.577
11	22.042	29.317	7.275	31.865	2.548	9.823
12	23.239	26.706	3.467	31.014	4.307	7.775
13	23.509	25.052	1.543	27.308	2.256	3.800
14	26.121	28.893	2.771	31.565	2.672	5.443
15	26.384	31.070	4.686	31.519	0.449	5.135
16	30.668	33.926	3.258	34.321	0.394	3.652
17	29.143	31.544	2.402	35.472	3.928	6.329
18	17.899	18.389	0.491	25.196	6.807	7.298
19	20.900	31.134	10.234	31.166	0.032	10.266
20	22.035	33.565	11.530	33.742	0.177	11.707
21	30.039	30.713	0.675	36.436	5.722	6.397
22	24.950	28.122	3.172	29.101	0.979	4.150
23	24.913	28.946	4.033	29.220	0.274	4.307
24	24.123	26.797	2.674	33.279	6.482	9.156

Table S8. Dataset on COVID-19 infections among travellers from Wuhan after continuity correction where symptom onset times and exposure window bounds are perturbed by a uniform random variable between 0 and 1. Column 1: individual index number. Columns 2-3: left and right bound, respectively, of the infecting exposure time. Column 4: exposure window. Column 5: symptom onset time. Columns 6-7: left and right bound, respectively, of the incubation period.

Transmission pair data on COVID-19

Downloaded from https://elifesciences.org/articles/65534/figures#content (8), the transmission pair data on COVID-19 has 14.823 days (observation number 20 in Table S9 below) as the largest observed right bound of the incubation period (after continuity correction). The latter reference assumes a Gamma distribution with shape 5.807 and scale 0.948 for the incubation distribution (based on the analysis of 9). For such a distribution, the 99th percentile is 12.2 days and we thus take t_u=20 for the upper bound of the incubation period. Based on previous studies on coronaviruses (see e.g. 6), we are quite confident that the range [0,20] (on which we specify our B-spline basis) overlaps with the region in the domain of the true underlying incubation density of COVID-19, above which the bulk of the probability mass is concentrated. Values in Table S9 can be reproduced from: https://github.com/oswaldogressani/Incubation/.

i	$t_i^{E_L}$	$t_i^{E_R}$	$\mathcal{E}_i$	t_i^S	$t_i^{\mathcal{I}_L}$	$t_i^{\mathcal{I}_R}$
1	50.225	51.085	0.860	54.433	3.348	4.208
2	50.325	51.757	1.432	54.802	3.045	4.477
3	52.345	52.601	0.256	56.479	3.878	4.134
4	52.639	52.661	0.022	56.983	4.322	4.344
5	52.317	52.768	0.450	56.528	3.761	4.211
6	53.658	53.920	0.262	59.186	5.266	5.528
7	53.882	53.953	0.071	59.734	5.781	5.852
8	55.028	55.929	0.901	59.374	3.445	4.346
9	55.045	55.201	0.156	59.921	4.720	4.876
10	49.131	49.709	0.578	55.744	6.035	6.613
11	49.234	49.819	0.586	55.661	5.841	6.427
12	51.967	53.216	1.249	60.808	7.592	8.841
13	56.133	56.412	0.279	61.775	5.363	5.642
14	19.861	24.640	4.779	25.623	0.983	5.762
15	26.286	33.267	6.981	36.837	3.570	10.551
16	26.265	26.305	0.039	27.456	1.151	1.191
17	26.259	26.992	0.733	33.201	6.209	6.942
18	32.622	34.330	1.708	35.312	0.982	2.690
19	32.504	35.494	2.990	36.153	0.659	3.649
20	40.042	46.317	6.275	54.865	8.548	14.823
21	44.121	44.893	0.771	49.565	4.672	5.443
22	45.384	46.070	0.686	48.519	2.449	3.135
23	49.143	49.544	0.402	55.472	5.928	6.329
24	53.900	55.134	1.234	55.166	0.032	1.266
25	51.204	53.134	1.930	53.280	0.147	2.076
26	54.039	55.713	1.675	62.436	6.722	8.397
27	48.913	48.946	0.033	49.220	0.274	0.307
28	47.869	50.503	2.634	50.632	0.129	2.763
29	47.229	49.082	1.854	49.439	0.357	2.210
30	52.429	52.576	0.146	56.555	3.979	4.126

31	50.431	51.073	0.642	56.637	5.565	6.206
32	51.709	54.427	2.718	56.584	2.157	4.875
33	56.639	56.993	0.354	60.743	3.750	4.104
34	56.835	59.733	2.898	59.822	0.089	2.987
35	56.732	59.308	2.575	60.526	1.219	3.794
36	54.280	58.185	3.905	60.566	2.381	6.286
37	51.701	52.479	0.778	54.639	2.159	2.938
38	52.494	52.797	0.303	54.509	1.712	2.014
39	55.487	55.768	0.281	65.731	9.963	10.245
40	55.835	55.978	0.142	59.330	3.352	3.494
41	78.002	78.108	0.106	82.206	4.098	4.204
42	80.985	81.947	0.962	82.193	0.246	1.208
43	81.167	81.923	0.756	82.653	0.729	1.486
44	81.416	81.777	0.361	82.622	0.846	1.206
45	57.908	61.344	3.437	63.662	2.318	5.754
46	67.930	68.272	0.342	74.440	6.167	6.509
47	26.590	26.815	0.225	33.736	6.921	7.145
48	41.555	45.880	4.324	50.989	5.109	9.433
49	36.776	36.998	0.222	45.255	8.256	8.479
50	39.607	41.574	1.967	49.247	7.673	9.640
51	35.517	41.175	5.658	41.392	0.216	5.875
52	36.572	44.141	7.568	45.625	1.485	9.053
53	56.308	56.657	0.349	56.913	0.256	0.605
54	56.835	60.248	3.412	60.332	0.084	3.497
55	58.681	58.865	0.184	61.990	3.126	3.309
56	58.420	58.551	0.131	61.060	2.509	2.640
57	33.429	39.389	5.960	39.561	0.171	6.132
58	55.939	60.835	4.895	68.560	7.726	12.621
59	30.285	37.465	7.180	37.710	0.245	7.426
60	29.411	29.639	0.229	43.116	13.477	13.706
61	35.971	37.117	1.146	48.976	11.859	13.005
62	34.249	34.886	0.637	37.836	2.950	3.587
63	34.033	34.648	0.614	38.615	3.967	4.582
64	34.300	34.888	0.587	37.799	2.911	3.499
65	34.217	34.689	0.471	35.290	0.601	1.073
66	34.520	34.705	0.185	37.676	2.971	3.156
67	38.196	38.928	0.733	45.561	6.633	7.365
68	38.080	38.374	0.294	50.441	12.068	12.362
69	35.871	36.945	1.073	47.803	10.859	11.932
70	31.086	31.509	0.423	32.101	0.592	1.015
71	31.605	35.378	3.772	35.746	0.369	4.141
72	68.457	73.107	4.651	73.390	0.282	4.933
73	73.063	77.684	4.621	77.868	0.183	4.804
74	68.493	69.856	1.363	76.641	6.785	8.148

Table S9. Dataset on transmission pairs for COVID-19 from (8) after continuity correction where symptom onset times and exposure window bounds are perturbed by a uniform random variable between 0 and 1. Column 1: individual index number. Columns 2-3: left and right bound, respectively, of the infecting exposure time. Column 4: exposure window. Column 5: symptom onset time. Columns 6-7: left and right bound, respectively, of the incubation period.

Data on Middle East Respiratory Syndrome

The dataset was obtained from the Supplementary Material (Table S2) of (10) (https://doi.org/10.1016/S1473-3099(13)70304-9) and consists of lower and upper bounds of the incubation period for seven individual MERS-CoV cases in the United Kingdom, France, Italy and Tunisia. Table S10 summarizes the data (after continuity correction). The latter reference fitted a lognormal distribution with a mean incubation period of 5.5 days and standard deviation (SD) of 2.5 days. From this information, the mean and SD of the natural logarithm of the distribution are easily calculated and given by the following values meanlog = 1.611 and sdlog = 0.433. The 99th percentile of the fitted lognormal distribution is thus given by 13.7 days. Also, in the analysis of (11), the 95th percentile of the fitted incubation distribution was reported to be equal to 12.4 days for MERS. Based on this information, we decide to choose t_u=20 for the upper bound of the incubation period to ensure that our B-spline basis covers a large part of the domain of the underlying incubation density. Values in Table S10 can be reproduced from the code available here: https://github.com/oswaldogressani/Incubation/.

i	$t_i^{\mathcal{I}_L}$	$t_i^{\mathcal{I}_R}$
1	6.114	9.622
2	1.609	4.623
3	9.861	12.64
4	3.009	4.233
5	4.283	4.923
6	3.292	3.837
7	4.187	4.232

Table S10. Dataset on Middle East Respiratory Syndrome (MERS) from (10) after continuity correction where incubation bounds are perturbed by a uniform random variable between 0 and 1. Column 1: individual index number. Column 2: left bound of the incubation period. Column 3: right bound of the incubation period.

Dataset on Mpox

The Mpox dataset was downloaded from the link https://github.com/fmiura/MpxInc_2022 provided in the Data availability statement of (12). It contains symptom onset times as well as the left and right bound, respectively, of infecting exposure windows. These quantities are perturbed by a uniform random variable between 0 and 1 to fit our model in continuous time and the resulting dataset is summarized in Table S11. The largest observed right bound of the incubation period (after continuity correction) is given by 18.840 days (see last column of Table S11 below). The parametric distributions (Weibull, Gamma and lognormal) fitted in (12) for the incubation period reveal an estimated 99th percentile of 20.3 days for the Weibull, 18.8 days for the Gamma and 23.3 days for the lognormal (the best-fitting distribution according to the leave-one-out information criterion). This motivates our conservative choice of t_u=30 for the upper bound of the incubation period, so that our B-spline basis specified in the range [0, 30] covers with high confidence the area of the domain of the true underlying incubation density that supports most of the probability mass. Values in Table S11 can be reproduced from the code available in the following repository: https://github.com/oswaldogressani/Incubation/.

i	$t_i^{E_L}$	$t_i^{E_R}$	$\mathcal{E}_i$	t_i^S	$t_i^{\mathcal{I}_L}$	$t_i^{\mathcal{I}_R}$
1	0.641	0.994	0.354	6.693	5.699	6.052
2	11.025	11.478	0.453	20.795	9.318	9.771
3	11.216	11.318	0.102	19.758	8.440	8.542
4	11.143	11.415	0.272	19.232	7.817	8.089
5	16.233	16.466	0.233	22.139	5.673	5.906
6	14.095	14.384	0.289	20.665	6.281	6.570
7	6.001	6.475	0.475	20.710	14.235	14.710
8	6.380	6.613	0.233	25.220	18.607	18.840
9	9.111	15.244	6.132	15.352	0.108	6.241
10	9.418	19.788	10.371	24.668	4.880	15.250
11	8.435	12.985	4.550	27.103	14.118	18.668
12	17.094	17.467	0.373	24.782	7.316	7.689
13	20.147	20.935	0.788	25.411	4.475	5.264
14	10.061	10.948	0.887	16.301	5.354	6.241
15	9.142	19.549	10.407	25.721	6.171	16.578
16	18.369	18.984	0.615	30.220	11.236	11.850
17	23.091	23.142	0.051	31.154	8.012	8.063
18	16.619	23.891	7.272	24.690	0.799	8.071

Table S11. Dataset on Mpox from (12) after continuity correction where symptom onset times and exposure window bounds are perturbed by a uniform random variable between 0 and 1. Column 1: individual index number. Columns 2-3: left and right bound, respectively, of the infecting exposure time. Column 4: exposure window. Column 5: symptom onset time. Columns 6-7: left and right bound, respectively, of the incubation period.

Mean, SD and quantiles of the estimated incubation periods for real datasets

Dataset origin	Distribution	Mean (days)		SD (days)	
		Estimate	95% CI	Estimate	95% CI
COVID-19 (6)	Lognormal	4.4	4.0-4.8	1.8	1.5-2.1
COVID-19 (8)	Weibull	4.5	4.2-4.9	3.1	2.9-3.4
MERS (10)	Lognormal	5.4	4.5-6.5	2.8	2.4-3.7
Mpox (12)	Lognormal	8.9	7.9-9.9	4.1	3.5-5.1

Table S12. Mean and SD of the estimated incubation periods with our flexible Bayesian approach for different datasets. CI: credible interval, SD: standard deviation.

Quantiles	Data on COVID-19 from (6)		Data on COVID-19 from (8)	
	Estimate	95% CI	Estimate	95% CI
$q_{0.05}$	2.2	1.7-2.6	0.7	0.5-0.8
$q_{0.25}$	3.2	2.6-3.6	2.2	1.9-2.5
$q_{0.50}$	4.1	3.6-4.6	3.9	3.5-4.3
$q_{0.75}$	5.3	4.9-5.8	6.2	5.8-6.7
$q_{0.95}$	7.7	7.2-8.5	10.5	9.8-11.4

Table S13. Quantiles of the estimated incubation periods with our flexible Bayesian approach for the COVID datasets. CI: credible interval.

Quantiles	Data on MERS from (10)		Data on Mpox from (12)	
	**Estimate**	**95% CI**	**Estimate**	**95% CI**
$q_{0.05}$	2.2	1.4-2.9	3.9	3.3-4.6
$q_{0.25}$	3.5	2.5-4.4	6.0	5.2-6.8
$q_{0.50}$	4.8	3.8-5.9	8.1	7.1-8.9
$q_{0.75}$	6.7	5.6-8.0	10.9	9.7-12.1
$q_{0.95}$	10.7	9.5-13.1	16.6	14.7-19.1

Table S14. Quantiles of the estimated incubation periods with our flexible Bayesian approach for the MERS and Mpox datasets. CI: credible interval.

References

1. Tierney, L. and Kadane, J. B. (1986). Accurate approximations for posterior moments and marginal densities. *Journal of the American Statistical Association*, **81**(393):82–86.

2. Rue, H., Martino, S., and Chopin, N. (2009). Approximate Bayesian inference for latent Gaussian models by using integrated nested Laplace approximations. *Journal of the Royal Statistical Society Series B*, **71**(2):319–392.

3. Schwarz, G. (1978). Estimating the dimension of a model. *The Annals of Statistics*, **6**(2):461–464.

4. Vehtari, A., Gelman, A., and Gabry, J. (2017). Practical Bayesian model evaluation using leave-one-out cross-validation and WAIC. *Statistics and Computing*, **27**(5):1413–1432.

5. Ferretti, L., Wymant, C., Kendall, et al. (2020). Quantifying SARS-CoV-2 transmission suggests epidemic control with digital contact tracing. *Science*, **368**(6491):eabb6936.

6. Backer, J. A., Klinkenberg, D., and Wallinga, J. (2020). Incubation period of 2019 novel coronavirus (2019-nCoV) infections among travellers from Wuhan, China, 20–28 January 2020. *Eurosurveillance*, **25**(5):2000062.

7. Donnelly, C. A., Ghani, A. C., Leung, G. M., et al. (2003). Epidemiological determinants of spread of causal agent of severe acute respiratory syndrome in Hong Kong. *The Lancet*, **361**(9371):1761–1766.

8. Hart, W. S., Maini, P. K., and Thompson, R. N. (2021). High infectiousness immediately before COVID-19 symptom onset highlights the importance of continued contact tracing. *Elife*, **10**:e65534.

9. Lauer, S. A., Grantz, K. H., Bi, Q., et al. (2020). The incubation period of coronavirus disease 2019 (COVID-19) from publicly reported confirmed cases: estimation and application. *Annals of Internal Medicine*, **172**(9):577–582.

10. Cauchemez, S., Fraser, C., Van Kerkhove, M. D., et al. (2014). Middle east respiratory syndrome coronavirus: quantification of the extent of the epidemic, surveillance biases, and transmissibility. *The Lancet Infectious Diseases*, **14**(1):50–56.

11. Assiri, A., McGeer, A., Perl, T. M., et al. (2013). Hospital outbreak of middle east respiratory syndrome coronavirus. *New England Journal of Medicine*, **369**(5), 407-416.

12. Miura, F., van Ewijk, C. E., Backer, J. A., et al. (2022). Estimated incubation period for monkeypox cases confirmed in the Netherlands, May 2022. *Eurosurveillance*, **27**(24):2200448.
